# hsa_circ_0007919 induces LIG1 transcription by binding to FOXA1/TET1 to enhance the DNA damage response and promote gemcitabine resistance in pancreatic ductal adenocarcinoma

**DOI:** 10.1186/s12943-023-01887-8

**Published:** 2023-12-04

**Authors:** Lei Xu, Xiao Ma, Xiuzhong Zhang, Chong Zhang, Yi Zhang, Shuai Gong, Nai Wu, Peng Zhang, Xinyu Feng, Jiaxuan Guo, Mengmeng Zhao, Zeqiang Ren, Pengbo Zhang

**Affiliations:** 1 Department of General Surgery, Affiliated Hospital of Xuzhou Medical University, Xuzhou, China; 2 Institute of Digestive Diseases, Xuzhou Medical University, Xuzhou, China; 3 Shandong First Medical University and Shandong Academy of Medical Sciences, Shandong Cancer Hospital and Institute, Jinan, China; 4 Department of General Surgery, Xuzhou First People’s Hospital, Xuzhou, China; 5 Department of General Surgery, Shangqiu Municipal Hospital, Shangqiu, China

**Keywords:** hsa_circ_0007919, LIG1, DNA damage repair, Pancreatic ductal adenocarcinoma, QKI

## Abstract

**Background:**

Circular RNAs (circRNAs) play important roles in the occurrence and development of cancer and chemoresistance. DNA damage repair contributes to the proliferation of cancer cells and resistance to chemotherapy-induced apoptosis. However, the role of circRNAs in the regulation of DNA damage repair needs clarification.

**Methods:**

RNA sequencing analysis was applied to identify the differentially expressed circRNAs. qRT-PCR was conducted to confirm the expression of hsa_circ_0007919, and CCK-8, FCM, single-cell gel electrophoresis and IF assays were used to analyze the proliferation, apoptosis and gemcitabine (GEM) resistance of pancreatic ductal adenocarcinoma (PDAC) cells. Xenograft model and IHC experiments were conducted to confirm the effects of hsa_circ_0007919 on tumor growth and DNA damage in vivo. RNA sequencing and GSEA were applied to confirm the downstream genes and pathways of hsa_circ_0007919. FISH and nuclear-cytoplasmic RNA fractionation experiments were conducted to identify the cellular localization of hsa_circ_0007919. ChIRP, RIP, Co-IP, ChIP, MS-PCR and luciferase reporter assays were conducted to confirm the interaction among hsa_circ_0007919, FOXA1, TET1 and the LIG1 promoter.

**Results:**

We identified a highly expressed circRNA, hsa_circ_0007919, in GEM-resistant PDAC tissues and cells. High expression of hsa_circ_0007919 correlates with poor overall survival (OS) and disease-free survival (DFS) of PDAC patients. Hsa_circ_0007919 inhibits the DNA damage, accumulation of DNA breaks and apoptosis induced by GEM in a LIG1-dependent manner to maintain cell survival. Mechanistically, hsa_circ_0007919 recruits FOXA1 and TET1 to decrease the methylation of the LIG1 promoter and increase its transcription, further promoting base excision repair, mismatch repair and nucleotide excision repair. At last, we found that GEM enhanced the binding of QKI to the introns of hsa_circ_0007919 pre-mRNA and the splicing and circularization of this pre-mRNA to generate hsa_circ_0007919.

**Conclusions:**

Hsa_circ_0007919 promotes GEM resistance by enhancing DNA damage repair in a LIG1-dependent manner to maintain cell survival. Targeting hsa_circ_0007919 and DNA damage repair pathways could be a therapeutic strategy for PDAC.

**Supplementary Information:**

The online version contains supplementary material available at 10.1186/s12943-023-01887-8.

## Introduction

As one of the most aggressive and deadly malignancies, pancreatic ductal adenocarcinoma (PDAC) has become the fourth leading cause of cancer-related death and is expected to advance to the second leading cause of cancer-related death within decades [1, 2]. Despite the advances in the understanding of the molecular mechanisms and development of therapies for PDAC over the past few decades, its 5-year survival rate remains the lowest among all malignancies [3]. There is continuous proliferative signal transduction during the development of PDAC, such continuous proliferation induced by oncogene expression could cause DNA replication stress, resulting in genomic instability and even apoptosis [4]. Therefore, cancer cells respond to DNA damage via activation of DNA damage response (DDR) pathways, mainly including base excision repair, nucleotide excision repair, mismatch repair, homologous recombination and non-homologous terminal junction [5]. During the cell cycle, more than 6 million DNA base pairs are replicated, and this process can be affected by many sources of damage and replication stress [6]. DNA repair endows tumors with potent genomic stability and antiapoptotic ability, which can easily promote the malignant progression of tumors [7]. In addition, various chemotherapies for PDAC can cause specific types of DNA damage; for example, platinum alkylating agents and the topoisomerase inhibitor irinotecan can lead to DNA double-strand breaks (DSB), and the antimetabolic drugs 5-fluorouracil (5-FU) and gemcitabine (GEM) can cause single-base damage and single-strand DNA breaks (SSBs)[8], which can develop into DSBs upon accumulation [9]. To date, PARP inhibitors and cell cycle checkpoint inhibitors have proven to be effective therapies for PDAC [10]. Studies on DDR mechanism defects and inhibitors of DNA damage repair may provide new insights for the treatment of PDAC.

CircRNAs are a class of noncoding RNAs and are single-stranded, circular, closed RNAs widely present in eukaryotic cells [11]. CircRNAs are formed from pre-mRNAs of their host genes through selective back-splicing and circularization, and they have high stability and cannot be easily degraded by RNA enzymes [12]. CircRNAs have been found to be involved in multiple steps of tumorigenesis and development, including DDR regulation [13]. CircSMARCA5 terminates SMARCA5 transcription at exon 15 to reduce its expression, thereby inhibiting SMARCA5-mediated DNA damage repair and cisplatin resistance in breast cancer (BC)[14]. CircITCH sponges miR-330-5p to increase SIRT6 expression, and SIRT6 then activates PARP1 to repair DNA damage to alleviate doxorubicin-induced cardiomyocyte damage and dysfunction [15]. However, there are few studies on the relationships between circRNAs and PDAC, and most of these have focused on miRNA-related research. Circ-MBOAT2 promotes the proliferation, metastasis and glutamine metabolism of PDAC cells through the miR-433-3p/GOT1 axis [16]. Cancer-associated fibroblast-specific circ-FARP1 binds to CAV1 and inhibits its ubiquitination by ZNRF1 to enhance the secretion of LIF; in addition, circ-FARP1 sponges miR-660-3p to increase the expression of LIF, thereby promoting the stemness and GEM resistance of PDAC cells [17]. To date, studies on the regulation of circRNAs on DNA damage in PDAC have not been reported.

In this study, we identified the highly expressed hsa_circ_0007919 in GEM-resistant PDAC tissues and cells. Hsa_circ_0007919 could inhibit the DNA damage and apoptosis induced by GEM chemotherapy and maintain cell survival. We found that mechanistically, hsa_circ_0007919 recruits FOXA1 and TET1 to decrease the methylation of the LIG1 promoter and enhance LIG1 transcription, then LIG1 involves in multiple DNA repair pathways to decrease GEM-related DNA damage. The function of hsa_circ_0007919 was also verified in xenograft model in nude mice.

## Materials and methods

### Clinical tissue samples

A total of 95 pairs of PDAC tissues and adjacent tumor tissues were collected from Xuzhou Medical University Affiliated Hospital. Among them, 50 patients had not received radiotherapy or chemotherapy while 45 patients received GEM neoadjuvant therapy. This study was approved by Institutional Ethics Committee of Xuzhou Medical University Affiliated Hospital and informed consent were signed by all patients.

### Cell culture and transfection

The normal human pancreatic duct cell line hTERT-HPNE and PDAC cell lines PANC-1, CFPAC-1, BxPC-3 and MIA-PaCa2 were purchased from Chinese Academy of Science (Shanghai, China) cultured RPMI 1640 medium (Hyclone, USA) containing 10% Fetal Bovine Serum (FBS) (Gibco,USA), 100u/ml penicillin and 100 µg/ml streptomycin (Beyotime, China) in a cell incubator at 37℃ with 5% CO_2_. For the construction of GEM-resistant PDAC cell lines, PANC-1 and CFPAC-1 cells were cultured with GEM (MCE, USA) at increasing concentration gradients. For 5-AzaC treatment, CRC cells were treated with 5µM of 5-AzaC (MCE, USA) for 72 h. All small interfering RNA (siRNA) and full-length plasmid of hsa_circ_0007919, LIG1, FOXA1, TET1 and negative control were purchased from GenePharma (Suzhou, China) and transfected into cells using Lipofectamine 2000 reagent (Invitrogen, USA) according to the manufacturer’s protocol. All sequences of siRNAs are shown as follows:

si-hsa_circ_0007919#1: 5’-GACAGAUCCAGGUGGAAGCTT-3’;

si-hsa_circ_0007919#2: 5’-ACAGAUCCAGGUGGAAGCATT-3’;

si-LIG1#1: 5’- AGAAGAUAGACAUCAUCAAAG-3’;

si-LIG1#2: 5’- CGUCAUUUCUUUCAAUAAAUA-3’;

si-FOXA1: 5’- GGAUGUUAGGAACUGUGAAGA-3’;

si-TET1: 5’- CGAAGCUACUGCAAAUCAACA-3’;

si-Ctrl: 5’- UUCUCCGAACGUGUCACGUTT-3’.

### RNA extraction and quantitative real-time PCR (qRT-PCR)

Total RNA was extracted from tissues and cells by RNA isolater Total RNA Extraction Kit (Vazyme, China), cDNA was synthesized by HiScript II Q RT SuperMix for qPCR (Vazyme, China) and the expression was detected by ChamQ SYBR qPCR Master Mix (Vazyme, China). All the data were normalized to GAPDH/U6 and the data from tissues were quantified by 2^−ΔCt^ method while others were quantified by 2^−ΔΔCt^ method. All the primers were synthesized by GENEray (Shanghai, China) and the sequences are shown as follows:

hsa_circ_0007919-F: 5’-AGGTGGAAGCAGGGAAAG-3’;

hsa_circ_0007919-R: 5’-TCATGGGCAGCAACAGG-3’;

ABR-F: 5’-GGTGGATTCCTTCGGCTAT-3’;

ABR-R: 5’-CACTTGGGCTCCGCTGT-3’;

LIG1-F: 5’-GCCCTGCTAAAGGCCAGAAG-3’;

LIG1-R: 5’-CATGGGAGAGGTGTCAGAGAG-3’;

FOXA1-F: GCAATACTCGCCTTACGGCT-3’;

FOXA1-R: TACACACCTTGGTAGTACGCC-3’;

TET1-F: CATCAGTCAAGACTTTAAGCCCT-3’;

TET1-R: CGGGTGGTTTAGGTTCTGTTT-3’;

LIG1 P1-F: GCTAAAACCTCCTCCCC-3’;

LIG1 P1-R: CATGAAGCATGTGACCG-3’;

GAPDH-F: 5’-GGAGCGAGATCCCTCCAAAAT-3’;

GAPDH-R: 5’-GGCTGTTGTCATACTTCTCATGG-3’;

U6-F: 5’-CTCGCTTCGGCAGCACA-3’;

U6-R: 5’-AACGCTTCACGAATTTGCGT-3’.

### Identification of hsa_circ_0007919

qPCR product amplified by hsa_circ_0007919 primer was validated by Sanger-seq (Sangon, China). Total gDNA was extracted by FastPure Cell/Tissue DNA Isolation Mini Kit (Vazyme, China) and qPCR products amplified from cDNA and gDNA were separated in 1% agarose gel. Total RNA was treated with RNase R (Epicentre, USA) at 37℃ for 30 min and were detected by qRT-PCR just as described above.

### Half maximal inhibitory concentration (IC50) detection assay

A total of 12 groups of 4 × 10^3^ PDAC cells were placed into 96-well plate separately, then cells were treated with GEM at concentration of 0, 0.1, 0.2, 0.4, 0.8, 1.6, 3.2, 6.4, 12.8, 25.6, 51.2 and 102.4µM for 48 h and then detected as CCK-8 assay described.

### CCK-8 assay

Cells after transfection or GEM treatment were collected and counted, then 4 × 10^3^ cells were placed into 96-well plate and cultured in incubator at 37℃. 24, 48, 72 and 96 h after, cells were incubated with 100 µl serum-free medium and 10 µl CCK-8 solution (Glpbio, USA) at 37℃ for 2 h and measured at 450 nm wavelength (SPARK, Switzerland).

### Apoptosis detection assay

Cells after transfection or GEM treatment were collected by EDTA-free trypsin solution (Beyotime, China), then cells were washed by PBS and incubate with Annexin V and PI solution for 10 min and detected by the flow cytometer (BD, USA) according to the manufacturer’s protocol of Cell Apoptosis Detection Kit (Biosharp, China).

### Western blot assay

Total protein was extracted from cells by RIPA lysis solution (Beyotime, China) and quantified by BCA Protein Assay Kit (Beyotime, China), protein was separated in SDS-PAGE and transferred to PVDF membrane (Millipore, Germany). After blocking with 5% skim milk, the membrane was incubated with primary antibodies and secondary antibodies and detected with Super ECL Detection Reagent (Yeasen, China) using a luminescent imaging system (Tanon, China). All used antibodies are shown as follows: anti-caspase3 (19677-1-AP, Proteintech, USA), anti-caspase9 (10380-1-AP, Proteintech, USA), anti-BCL2 (68103-1-Ig, Proteintech, USA), anti-GAPDH (60004-1-Ig, Proteintech, USA), HRP-goat anti-rabbit IgG (H + L) (BF03008, Biodragon, China), HRP-goat anti-mouse IgG (H + L) (BF03001, Biodragon, China), anti-γ-H2AX (AP0687, Abclonal, China), CoraLite594-conjugated Goat Anti-Rabbit IgG (H + L) (SA00013-4, Proteintech, USA), anti-LIG1 (18051-1-AP,, Proteintech, USA), anti-Ki67 (GB111499, Servicebio, China), anti-FOXA1 (GTX100308, GeneTex, USA), anti-TET1 (AB_2793752, Active Motif, USA), anti-QKI (13169-1-AP, Proteintech, USA).

### Single cell gel electrophoresis

0.8% normal melting point agarose (Vicmed, China) was placed on glass slide, then 5 × 10^3^ cells in 0.6% low melting point agarose (Biosharp, China) was place above and electrophoreted in a horizontal electrophoresis tank after lysis, at last cells were stained with PI solution (Biosharp, China) and the picture was photographed by inverted fluorescence microscope (Olympus, Japan).

### DNA ladder assay

Total DNA of cells after transfection was extracted by FastPure Cell/Tissue DNA Isolation Mini Kit (Vazyme, China). Briefly, cells were collected and treated by RNase Solution and Proteinase K at room temperature. Then cells were mixed with buffer GB and anhydrous ethanol, after abstersion with washing buffer, DNA was dissolved into elution buffer. At last, DNA was separated in 1% agarose gel and photographed using luminescent imaging system (Tanon, China).

### Immunofluorescence (IF)

Cells were fixed by 4% paraformaldehyde (Vicmed, China) and blocked by 5% Bovine Serum Albumin (BSA) (Solarbio, China), then cells were incubated with primary antibody, fluorescent secondary antibody and DAPI (Bioss, China) and photographed by confocal laser microscope (ZEISS, Germany).

### Stable inhibition cell lines construction and xenograft model

PANC-1 and CFPAC-1 GEM-resistant cells were infected by hsa_circ_0007919 inhibition lentivirus (GenePharma, China) or negative control lentivirus and selected by puromycin (Solarbio, China) for over 2 weeks, the efficiency of lentivirus was detected by qPCR. 5 × 10^6^ lentivirus-infected cells were injected into blank region of nude mice (Gempharmatech, China) and were treated with GEM (50 mg/kg, i.p.) every 4 days. After measuring volumes of tumors every 5 days, the mice were sacrificed and the tumors were harvested 25 days after injection. All sequences of shRNAs are shown as follows:

sh-hsa_circ_0007919: 5’-CACCGAGGTGGAAGCAGGGAAAGTTCGA AAAAATTGATCAATGCCGAGGA-3’;

sh-Ctrl: 5’-CACCGTTCTCCGAACGTGTCACGTTTCGAAAAACGTG ACACGTTCGGAGAA-3’.

### Immunohistochemical (IHC)

Tumors were fixed by 4% paraformaldehyde, paraffin embed and sliced into sections, sections were hydrated by xylene and gradient alcohol (Sinoreagent, China). Antigen of sections were repaired by citrate solution and blocked by goat serum, then sections incubated with primary and secondary antibodies according to the manufacturer’s protocol of SP Kit (ZSGB-BIO, China) and stained by DAB Staining Kit (ZSGB-BIO, China) and hematoxylin solutions (Sinoreagent, China). The pictures of sections were photographed by inverted microscope (Olympus, Japan). Relative staining score was calculated using an IHC score analysis method according to the proportion of positively stained cells and the intensity of staining. The proportion of positive cells was scored as follows: 0 (0–5%), 1 (6–25%), 2 (26–50%), 3 (51–75%), 4 (> 75%) and the intensity was scored as follows: 0 (negative), 1 (weak), 2 (moderate), 3 (strong).

### TUNEL assay

The TUNEL assay was performed according to the manufacturer’s protocol of TUNEL Apoptosis Detection Kit (Color Development) (Beyotime, China). Tissue sections was hydrated as described in IHC, after treated with Proteinase K at 37℃, the tissues were incubated with 3% hydrogen peroxide solution and biotin labeling solution containing TdT enzyme and biotein-dUDP away from light at 37℃, then the tissues were stained using Streptavidin-HRP solution and DAB staining solution and photographed by inverted microscope (Olympus, Japan).

### Gene set enrichment analysis (GSEA)

GSEA was performed on the normalized data using the GSEA v2.0 tool (http://www.broad.mit.edu/gsea/). We compared the expression of genes in PANC-1 GEM-resistant cells transfected with hsa_circ_0007919 siRNA or negative control siRNA. Three gene sets were used for analysis (KEGG_BASE_EXCISION_REPAIR, KEGG_MISMATCH_REPAIR, KEGG_NUCLEOTIDE_EXCISION_REPAIR), and the detailed genes in the gene sets can be found in MSigDB (http://software.broadinstitute.org/gsea/msigdb/genesets.jsp). The P values of the differences between the two gene sets were analyzed with the Kolmogorov–Smirnov test.

### Fluorescence in situ hybridization (FISH)

Cells were fixed by 4% paraformaldehyde and incubated with hybridization solution containing probes at 37℃ overnight, then cells were washed by SSC solution and stained with DAPI according to the manufacturer’s protocol of FISH Kit (RIBOBIO, China). The images were photographed by confocal laser microscope (ZEISS, Germany).

### Nuclear-cytoplasmic fractionation assay

Nuclear and cytoplasmic RNA was extracted by Cytoplasmic & Nuclear RNA Purification Kit (Norgen Biotek, Canada) according to the manufacturer’s protocol, then the expression of hsa_circ_0007919 in nucleus and cytoplasm was detected by qPCR.

### Chromatin isolation by RNA purification (ChIRP)

ChIRP was used to detect the protein binding with hsa_circ_0007919 and performed according to the manufacturer’s protocol of ChIRP kit (Bersinbio, China). Briefly, cells were cross-linked with paraformaldehyde and lysed through sonication, and then the lysis solution was incubated with biotin-labeled hsa_circ_0007919 probes (RIBOBIO, China) and magnetic beads, ultimately the protein was extracted and detected by WB.

### Co-immunoprecipitation (Co-IP)

Total protein extracted by cell lysis buffer for IP (Beyotime, China) was incubated with antibodies and magnetic beads, binding proteins were extracted by 2×SDS-PAGE Sample Loading Buffer (Beyotime, China) and detected by WB.

### RNA binding protein immunoprecipitation (RIP)

RIP assay was conducted according to the manufacturer’s protocol of RNA Immunoprecipitation Kit (GENESEED, China), mixture of RNA and protein was collected and incubated with antibodies and magnetic beads, RNA was extracted by adsorption column and detected by qPCR.

### Chromatin immunoprecipitation (ChIP)

ChIP assay was conducted according to the manufacturer’s protocol of Simple ChIP Enzymatic Chromatin IP Kit (CST, USA). Cells were mixed by 1% paraformaldehyde and chromatin was digested into fragments by enzymes. Then the solution was incubated with antibodies and magnetic beads and DNA was extracted from beads by purified centrifugal column, the binding DNA fragments were detected by qPCR.

### Methylation-specific PCR (MS-PCR)

Total DNA was extracted by FastPure Cell/Tissue DNA Isolation Mini Kit (Vazyme, China), then DNA was denaturated and bisulfite convered by EZ DNA Methylation-Gold Kit (ZYMO RESEARCH, USA) and amplificated by Methylation Specific PCR Kit (TIANGEN, China) according to the manufacturer’s protocol, the DNA was ultimately separated in 1% agarose gel and photographed by luminescent imaging system. Methylated primer and unmethylated primer located in LIG1 promoter were generated by MethPrimer 2.0 (http://www.urogene.org/methprimer2/) and the sequences are shown as follows:

LIG1 M-F: 5’-GAGAAGAAGGTTCGTTTTCGTAG-3’;

LIG1 M-R: 5’-ATAAAATAAATAAAATACCCCGAAT-3’;

LIG1 U-F: 5’-GAGAAGAAGGTTTGTTTTTG-3’;

LIG1 U-R: 5’-AAAATAAAATAAATAAAATACCCCAAAT-3’.

### Luciferase reporter assay

Luciferase reporter assay was conducted according to the manufacturer’s protocol of Dual Luciferase Reporter Gene Assay Kit (Beyotime, China). Cells transfected with pGL3-basic plasmid containing LIG1 promoter (Genecreate, China) and pRL-TK control plasmid with or without hsa_circ_0007919 inhibition were collected for lysis, then the firefly luciferase detection reagent and renilla luciferase detection reagent was added into the solution and measured by Multifunctional microplate reader (SPARK, Switzerland) separately.

### Statistical analysis

All values are expressed as mean ± standard deviation (SD). The significance of the differences was measured by Student’s t-test or one-way ANOVA. Kaplan–Meier analysis was used for survival analysis, and the differences of survival probabilities were measured by the log-rank test. The correlations between the expression of hsa_circ_0007919 and various clinicopathological variables were analyzed by Chi-Squared test. *p* < 0.05 was considered significant. Statistical analyses were performed using SPSS version 25.0 (SPSS, Inc., USA).

## Results

### hsa_circ_0007919 is upregulated in GEM-resistant PDAC and predicts poor prognosis

**Fig. 1:**
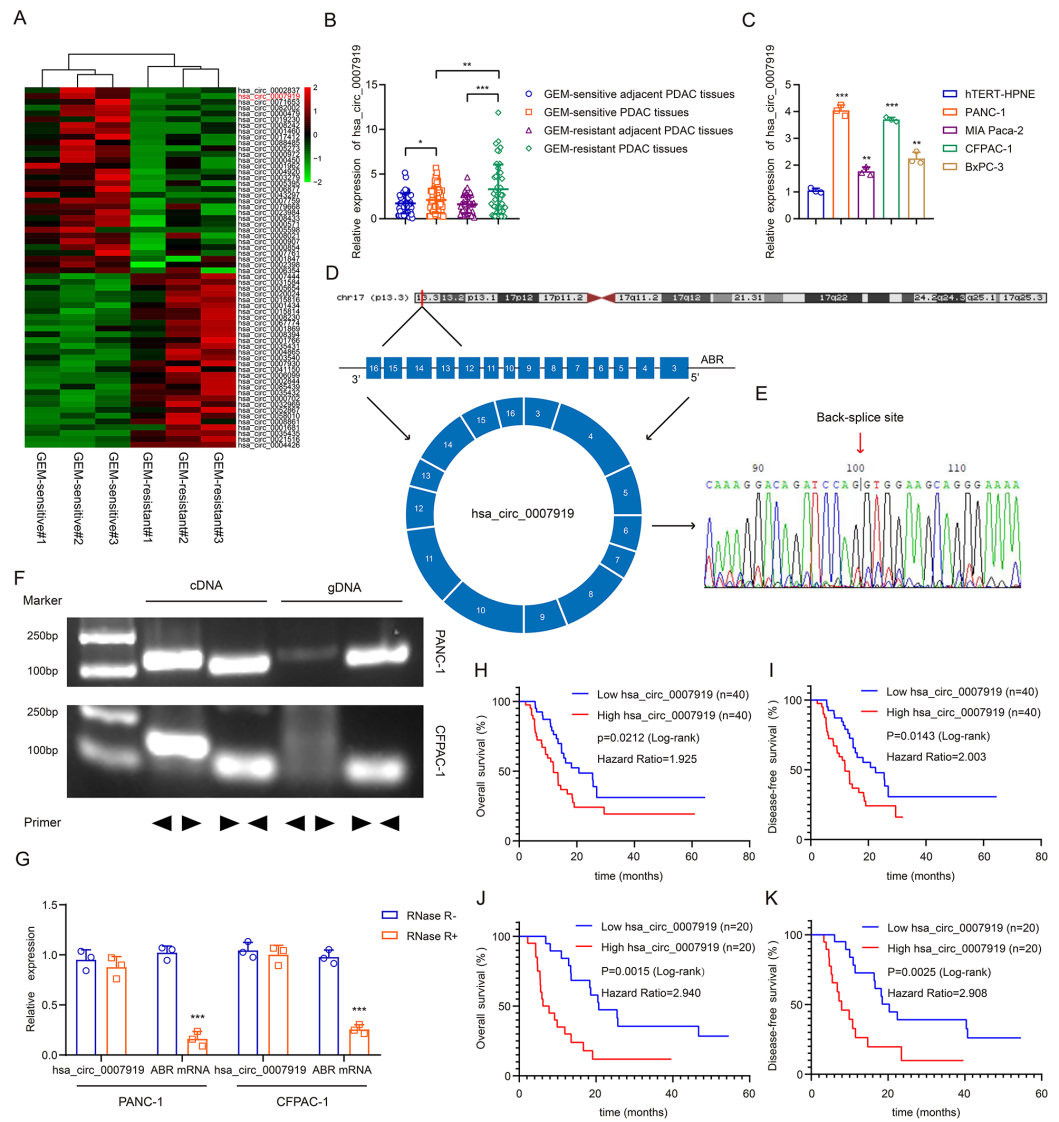
hsa_circ_0007919 is upregulated in GEM-resistant PDAC and predicts poor prognosis **(A)** Hierarchical clustering showing differentially expressed circRNAs in GEM-sensitive and GEM-resistant PDAC tissues (FC > 1 or <-1, *p* < 0.05). **(B)** The relative expression of hsa_circ_0007919 in GEM-sensitive and GEM-resistant PDAC tissues and corresponding adjacent PDAC tissues. **(C)** The relative expression of hsa_circ_0007919 in PDAC cells and normal pancreatic cells. **(D)** The genomic location and back-splicing of hsa_circ_0007919. **(E)** The splicing site of hsa_circ_0007919 validated by Sanger-seq. **(F)** PCR and agarose gel electrophoresis analysis of the presence of hsa_circ_0007919 and ABR in cDNA and gDNA samples from PDAC cells. **(G)** Expression of hsa_circ_0007919 and ABR in PDAC cells with or without RNase R treatment. **(H-I)** Kaplan–Meier analysis of the OS rate and DFS rate in PDAC patients with high or low expression of hsa_circ_0007919. **(J-K)** Kaplan–Meier analysis of the OS rate and DFS rate in GEM-resistant PDAC patients with high or low expression of hsa_circ_0007919. Data are the means ± SDs (n = 3 independent experiments), * *p* < 0.05, ** *p* < 0.01, *** *p* < 0.001

We performed next-generation sequencing to identify circRNAs that contribute to GEM resistance in three GEM-resistant PDAC tissues and three GEM-sensitive PDAC tissues. A total of 62 circRNAs were differentially expressed (FC<-1 or > 1 and *p* < 0.05), and hsa_circ_0007919 was significantly upregulated in GEM-resistant PDAC tissues compared with GEM-sensitive tissues (log2FC = 4.213454, *p* = 0.000312, Fig. 1A). Then, we measured the expression of hsa_circ_0007919 in 50 pairs of non-GEM-resistant PDAC tissues and adjacent tissues and 45 pairs of GEM-resistant PDAC tissues and related adjacent tissues. The results showed that the expression of hsa_circ_0007919 was markedly upregulated in GEM-resistant PDAC tissues compared with GEM-sensitive PDAC tissues (Fig. 1B). Compared with that in normal human pancreatic duct cells, the expression of hsa_circ_0007919 was increased in PDAC cells, including PANC-1, CFPAC-1, BxPC-3 and MIA-Paca2, and its expression level was relatively high in PANC-1 and CFPAC-1 cells (Fig. 1C). We next evaluated the structure of hsa_circ_0007919, which is derived from exons 3–16 of the ABR gene, and validated the circularization site of hsa_circ_0007919 by Sanger-seq (Fig. 1D-E); the results were consistent with the data obtained from the circBase database (https://www.circbase.org). We also designed divergent and convergent primers to detect the expression of hsa_circ_0007919 in both cDNA and gDNA. The results showed that hsa_circ_0007919 could be amplified from cDNA but not gDNA (Fig. 1F), and the resistance of hsa_circ_0007919 to digestion by the RNase R exonuclease confirmed that it was indeed circular (Fig. 1G). At last, we explored data from clinical tissue samples to analyze the correlations between hsa_circ_0007919 expression and clinicopathological features. We divided the 80 PDAC patients with or without GEM treatment into two groups with high expression (40 samples) or low expression (40 samples) of hsa_circ_0007919. As shown in Table 1, high expression of hsa_circ_0007919 was significantly correlated with vascular invasion (*p* = 0.032), nerve invasion (*p* = 0.039), T stage (*p* = 0.018), lymph node metastasis (*p* = 0.034) and TNM stage (*p* = 0.003), while there was no prominent association of hsa_circ_0007919 expression with age, gender, tumor location, degree of differentiation. Moreover, analysis of the relationship of hsa_circ_0007919 expression with overall survival (OS) and disease-free survival (DFS) of GEM-resistant patients showed that high expression of hsa_circ_0007919 predicted poor OS and DFS (*p* < 0.05) (Fig. 1H-I). Furthermore, we divided the 40 GEM-treated PDAC patients into two groups with high expression (n = 20) or low expression (n = 20) of hsa_circ_0007919 and found that high expression of hsa_circ_0007919 similarly predicted poor OS and DFS (*p* < 0.01) (Fig. 1J-K).

**Table 1:** Relationships between hsa_circ_0007919 expression and the clinicopathological characteristics of PDAC patients

Clinicopathological	Cases	hsa_circ_0007919 level			
parameters	80	High(40)	Low(40)	χ^2^	p-value
Age^a^				0.220	0.639
< 60	28	13	15		
≥ 60	52	27	25		
Gender^a^				0.549	0.459
Female	23	13	10		
Male	57	27	30		
Tumor location^a^				0.879	0.348
Head	52	28	24		
Body and tail	28	12	16		
Vascular invasion^a^				4.588	0.032*
Yes	62	35	27		
No	18	5	13		
Nerve invasion^a^				4.267	0.039*
Yes	60	34	26		
No	20	6	14		
Degree of differentiation^b^				2.453	0.313
Highly	2	0	2		
Moderately	27	12	15		
Poorly	51	28	23		
T stage^b^				9.895	0.018*
T1	9	1	8		
T2	33	14	19		
T3	20	13	7		
T4	18	12	6		
Lymph node metastasis^b^				7.296	0.034*
N0	36	12	24		
N1	34	22	12		
N2	10	6	4		
TNM stage^a^				11.958	0.003**
I	24	5	19		
II	32	19	13		
III	24	16	8		

^a^ Pearson chi-square test; ^b^ Fisher’s exact test. *P < 0.05, **P < 0.01

TNM staging is classified according to the 8th edition of the American Joint Committee on Cancer (AJCC).

### hsa_circ_0007919 inhibits DNA damage and gemcitabine sensitivity

**Fig. 2:**
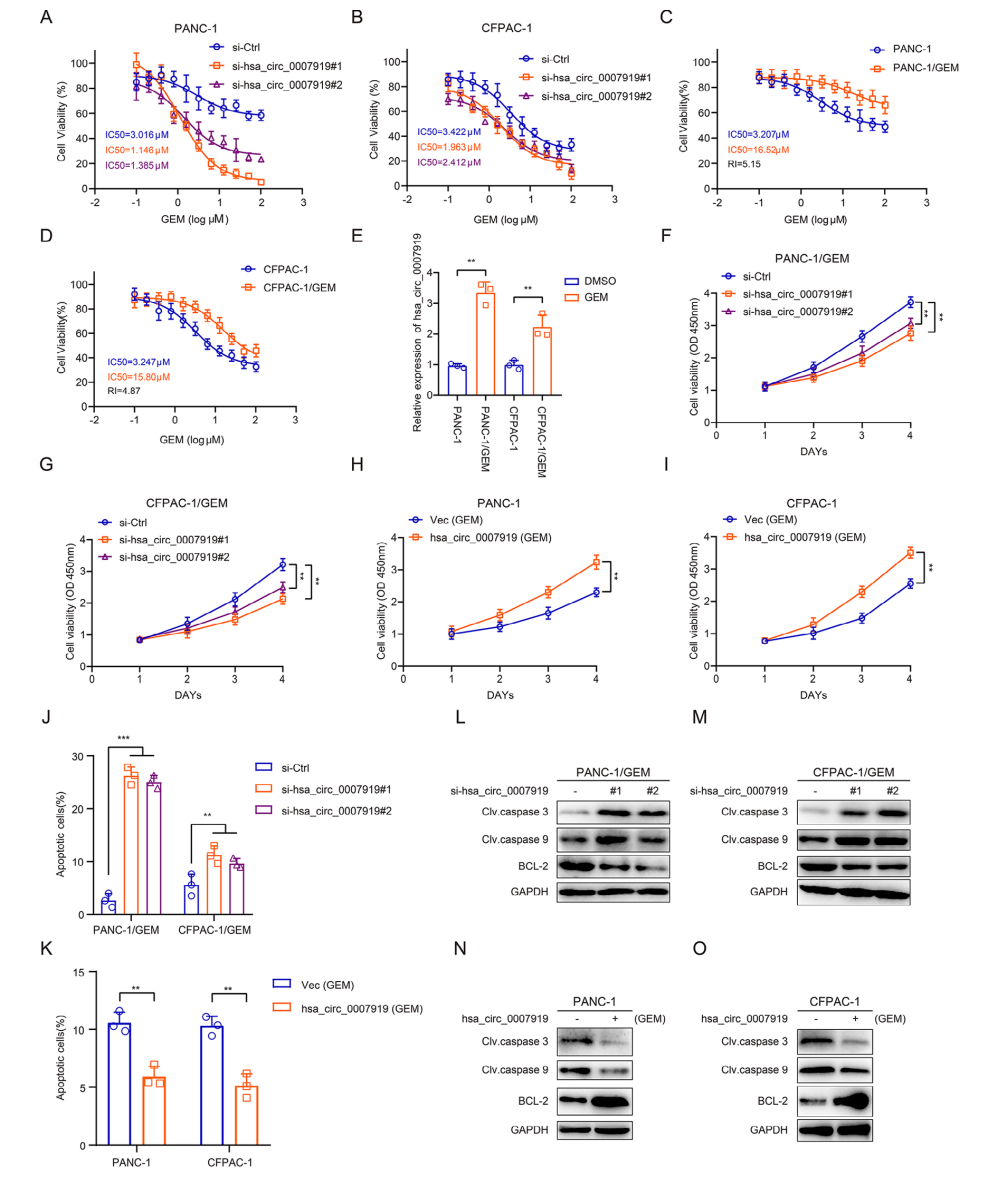
hsa_circ_0007919 inhibits gemcitabine sensitivity and apoptosis of GEM-resistant PDAC cells **(A-B)** CCK-8 analysis of the sensitivity of PDAC cells with or without hsa_circ_0007919 inhibition under different concentrations of GEM. **(C-D)** CCK-8 analysis of the sensitivity of normal and GEM-resistant PDAC cells under different concentrations of GEM. **(E)** Expression of hsa_circ_0007919 in normal and PDAC-resistant PDAC cells. **(F-G)** CCK-8 analysis of the proliferation of GEM-resistant PDAC cells with or without hsa_circ_0007919 inhibition. **(H-I)** CCK-8 analysis of the proliferation of PDAC cells with or without hsa_circ_0007919 overexpression under GEM treatment condition. **(J)** Flow cytometry analysis of the apoptotic rate of PDAC-resistant PDAC cells with or without hsa_circ_0007919 inhibition. **(K)** Flow cytometry analysis of the apoptotic rate of PDAC PDAC cells with or without hsa_circ_0007919 overexpression under GEM treatment condition. **(L-M)** Expression of apoptosis-related proteins in GEM-resistant PDAC cells with or without hsa_circ_0007919 inhibition. **(N-O)** Expression of apoptosis-related proteins in GEM PDAC cells with or without hsa_circ_0007919 overexpression under GEM treatment condition. Data are the means ± SDs (n = 3 independent experiments), ** *p* < 0.01, *** *p* < 0.001

**Fig. 3:**
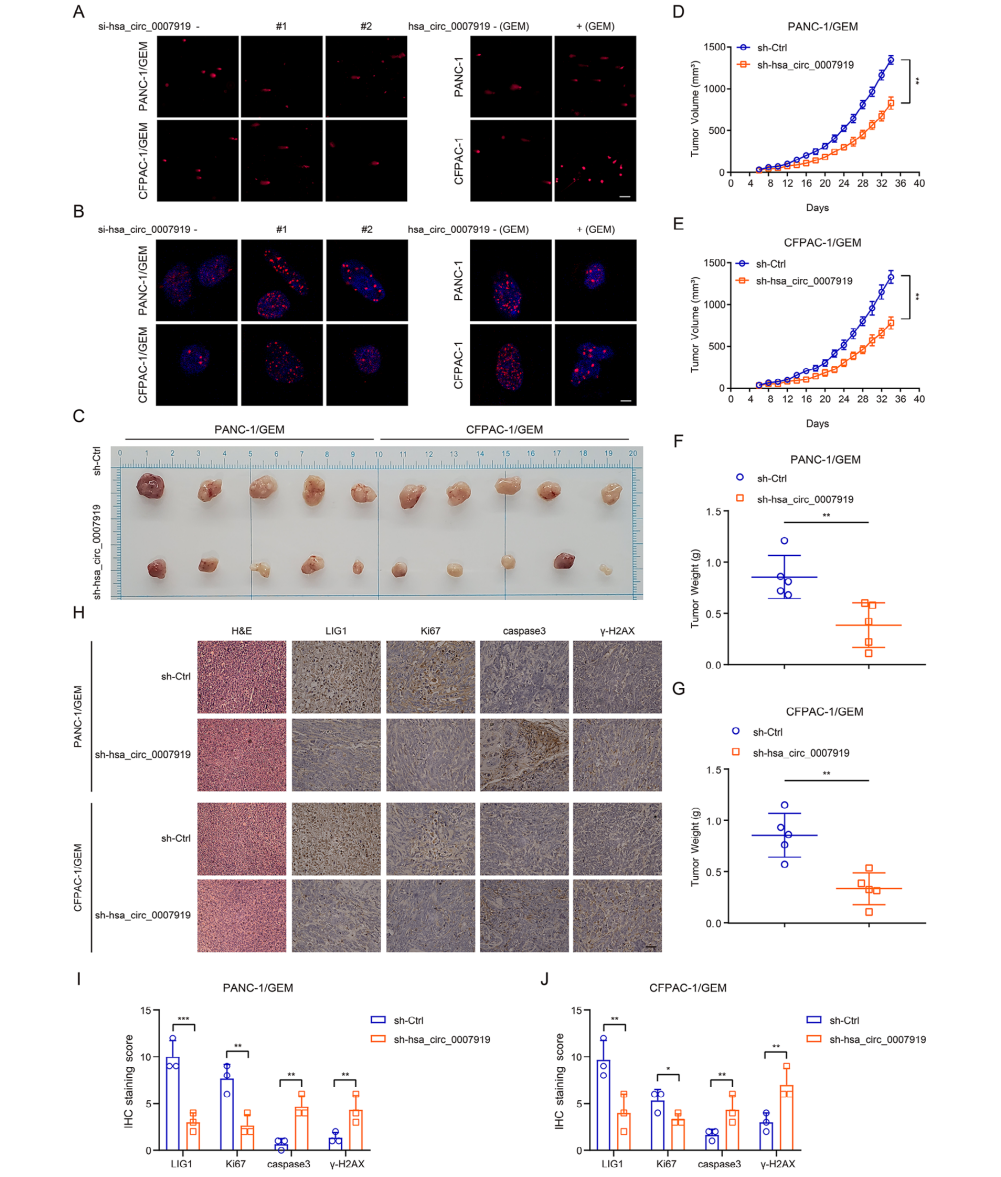
hsa_circ_0007919 inhibits DNA damage of GEM-resistant PDAC cells and proliferation of tumor in vivo **(A)** Comet analysis of the DNA damage of GEM-resistant PDAC cells with or without hsa_circ_0007919 inhibition and of the DNA damage of PDAC cells with or without hsa_circ_0007919 overexpression under GEM treatment condition (200×). **(B)** IF analysis of γ-H2AX accumulation in GEM-resistant PDAC cells with or without hsa_circ_0007919 inhibition and of γ-H2AX accumulation in PDAC cells with or without hsa_circ_0007919 overexpression under GEM treatment condition (1000×). **(C)** Image of tumors formed by GEM-resistant PDAC cells with or without hsa_circ_0007919 inhibition (n = 5). **(D-E)** Growth curves of tumors formed by GEM-resistant PDAC cells with or without hsa_circ_0007919 inhibition (n = 5). **(F-G)** Weights of tumors formed by GEM-resistant PDAC cells with or without hsa_circ_0007919 inhibition (n = 5). **(H)** IHC analysis of the expression of LIG1, Ki67, caspase 3 and γ-H2AX in tumors formed by GEM-resistant PDAC cells with or without hsa_circ_0007919 inhibition (400×). **(I-J)** IHC staining score analysis of images from Fig. 3H. Data are the means ± SDs (n = 3 independent experiments), ** *p* < 0.01

GEM is one of the common chemotherapy drugs in the clinical treatment of PDAC and can cause SSBs and DSBs by mediating base damage; however, PDAC patients often have adverse clinical outcomes due to chemoresistance [18]. Since hsa_circ_0007919 was upregulated in GEM-resistant PDAC tissues, we investigated its function in GEM-resistant cells. Firstly, we silenced hsa_circ_0007919 in PDAC cells and found that hsa_circ_0007919 inhibition increased GEM sensitivity (Fig. 2A-B, S1A). Then, we constructed GEM-resistant PDAC cell lines, PANC-1/GEM and CFPAC-1/GEM (Fig. 2C-D), and found that hsa_circ_0007919 was highly expressed in these GEM-resistant cells (Fig. 2E). Once again, we silenced hsa_circ_0007919 in both of these GEM-resistant cell lines and overexpressed it in normal PANC-1 and CFPAC-1 cells treated with GEM (Fig. S1B-C), and the results of the CCK-8 assay, FCM assay and DNA Ladder assay indicated that hsa_circ_0007919 silencing decreased the proliferation and increased the apoptosis of cells, while hsa_circ_0007919 overexpression had the opposite effects (Fig. 2F-K, S1D-G). Consistent with the apoptosis assay results, hsa_circ_0007919 silencing increased the levels of cleaved caspase 3 and cleaved caspase 9 and decreased BCL2 expression, while hsa_circ_0007919 overexpression decreased the levels of cleaved caspase 3 and cleaved caspase 9 and increased BCL2 expression (Fig. 2L-O). GEM, which functions as a pyrimidine antimetabolic agent, can induce single-base damage and lead to DNA breaks, so we evaluated the influence of hsa_circ_0007919 on DNA damage and found that hsa_circ_0007919 silencing increased the tail of single cells in gel electrophoresis and the accumulation of γ-H2AX in the nucleus, while hsa_circ_0007919 overexpression decreased these parameters (Fig. 3A-B). At last, we established xenograft model in nude mice and found that the volume and weight of tumors formed by hsa_circ_0007919-silenced PANC-1/GEM and PANC-1/GEM cells were decreased compared with those of tumors formed by control cells (Fig. 3C-G). The IHC, TUNEL assay and qPCR results showed that hsa_circ_0007919 silencing decreased the expression of Ki-67 and increased the expression of caspase3 and γ-H2AX and cell apoptosis (Fig. 3H-J, S2A-B). These results revealed that hsa_circ_0007919 enhances GEM resistance in PDAC cells by decreasing DNA damage to promote proliferation and reduce apoptosis.

### hsa_circ_0007919 inhibits DNA damage through LIG1-mediated repair pathways

**Fig. 4:**
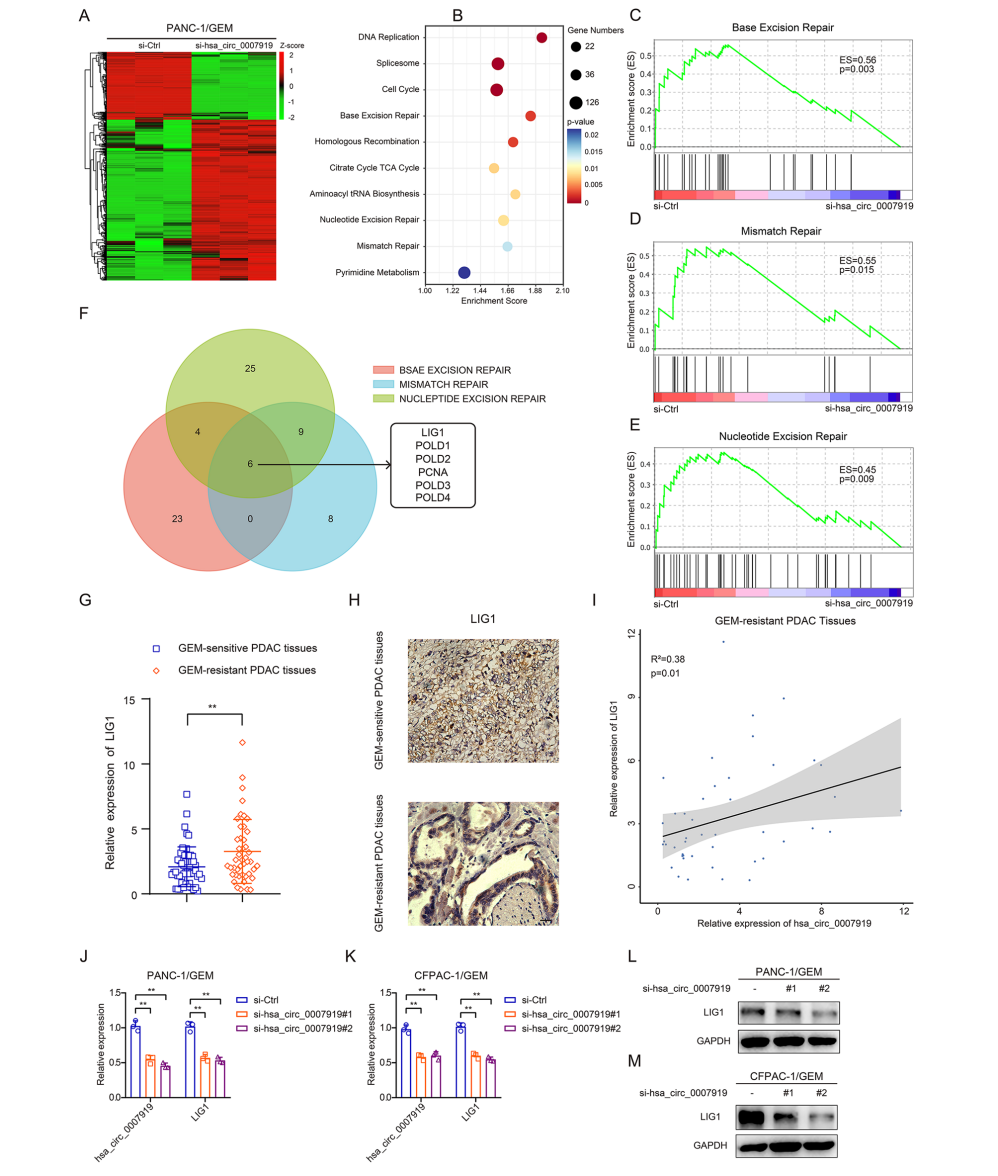
hsa_circ_0007919 inhibits DNA damage through LIG1-mediated repair pathways **(A)** Hierarchical clustering showing differentially expressed genes in GEM-resistant PDAC cells with or without hsa_circ_0007919 inhibition (FC > 1 or < -1, *p* < 0.05). **(B)** KEGG enrichment analysis of hsa_circ_0007919-regulated gene expression events. **(C-E)** GSEA enrichment analysis of hsa_circ_0007919-regulated gene expression events. **(F)** Venn diagram showing overlapped genes between differentially expressed genes from base excision repair, mismatch repair and nucleotide excision repair pathways. **(G-H)** Expression of LIG1 in GEM-sensitive and -resistant PDAC tissues at mRNA and protein level (400×). **(I)** Correlation analysis of hsa_circ_0007919 and LIG1 expression in GEM-resistant tissues. **(J-M)** Expression of LIG1 in GEM-resistant cells with or without hsa_circ_0007919 inhibition at mRNA and protein level. Data are the means ± SDs (n = 3 independent experiments), ** *p* < 0.01

To confirm how hsa_circ_0007919 inhibits DNA damage and influences GEM sensitivity, we performed RNA-seq to identify the differentially expressed genes in hsa_circ_0007919-silenced PANC-1/GEM cells compared with control cells. There were 520 upregulated and 219 downregulated genes (Fig. 4A), and KEGG analysis and GSEA showed that these genes were enriched in multiple DNA damage repair pathways, including base excision repair, mismatch repair and nucleotide excision repair (Fig. 4B-E). LIG1 was the most significantly downregulated gene common to all of these pathways (Fig. 4F, S2C-E). LIG1, a member of the DNA ligase family, has been reported to play an important role in DNA recombination in almost all DNA damage repair pathways [19]. Therefore, we measured the expression of LIG1 and found that it was also highly expressed in GEM-resistant PDAC tissues compared with normal PDAC tissues and was positively correlated with the expression of hsa_circ_0007919 in PDAC tissues (Fig. 4G-I). The mRNA and protein expression levels of LIG1 were decreased after hsa_circ_0007919 silencing and increased when hsa_circ_0007919 was overexpressed (Fig. 4J-M). These results revealed that hsa_circ_0007919 induces LIG1 expression to activate DNA damage repair pathways and enhance resistance to GEM in PDAC cells.

### LIG1 reversed the effects of hsa_circ_0007919 on cell proliferation, apoptosis and DNA damage

**Fig. 5:**
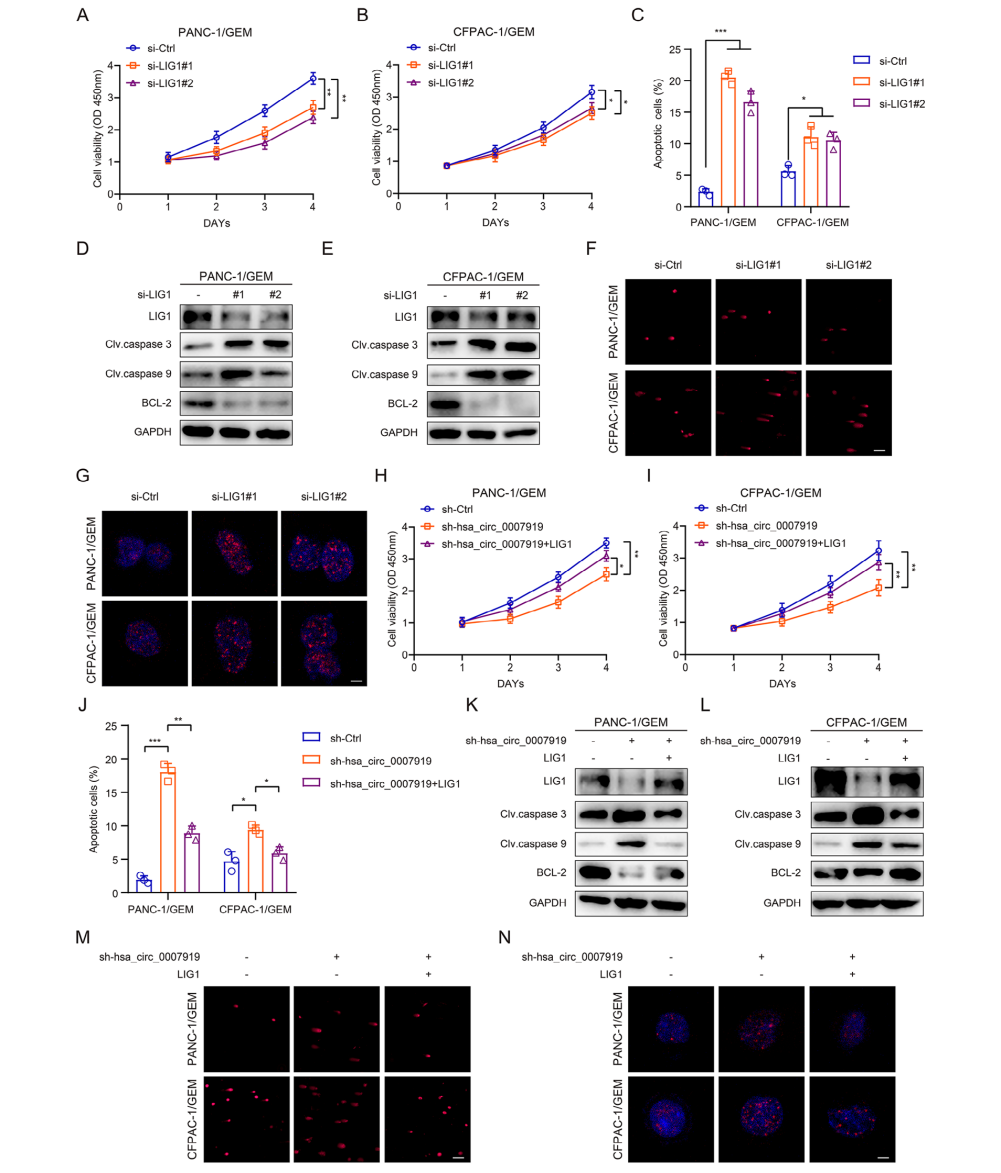
LIG1 reversed cell proliferation, apoptosis and DNA damage effects of hsa_circ_0007919 **(A-B)** CCK-8 analysis of the proliferation of GEM-resistant PDAC cells with or without LIG1 inhibition. **(C)** Flow cytometry analysis of the apoptotic rate of PDAC-resistant PDAC cells with or without LIG1 inhibition. **(D-E)** Expression of apoptosis-related proteins in GEM-resistant PDAC cells with or without LIG1 inhibition. **(F)** Comet analysis of the DNA damage of GEM-resistant PDAC cells with or without LIG1 inhibition (200×). **(G)** IF analysis of γ-H2AX accumulation in GEM-resistant PDAC cells with or without LIG1 inhibition (1000×). **(H-I)** CCK-8 analysis of the proliferation of hsa_circ_0007919-inhibited GEM-resistant PDAC cells with or without LIG1 overexpression. **(J)** Flow cytometry analysis of the apoptotic rate of hsa_circ_0007919-inhibited PDAC-resistant PDAC cells with or without LIG1 overexpression. **(K-L)** Expression of apoptosis-related proteins in hsa_circ_0007919-inhibited GEM-resistant PDAC cells with or without LIG1 overexpression. **(M)** Comet analysis of the DNA damage of hsa_circ_0007919-inhibited GEM-resistant PDAC cells with or without LIG1 overexpression (200×). **(N)** IF analysis of γ-H2AX accumulation in hsa_circ_0007919-inhibited GEM-resistant PDAC cells with or without LIG1 overexpression (1000×). Data are the means ± SDs (n = 3 independent experiments), * *p* < 0.05, ** *p* < 0.01, *** *p* < 0.001

To confirm that LIG1 is the downstream target of hsa_circ_0007919, we investigated the role of LIG1 in GEM-resistant PDAC cells and found that silencing LIG1 resulted in decreased proliferation and increased apoptosis and DNA damage (Fig. 5A-G, S3A-B). Moreover, we further overexpressed LIG1 in PANC-1/GEM and PANC-1/GEM cells with stable hsa_circ_0007919 silencing (Fig. S3C-D) and found that LIG1 overexpression reversed cell proliferation, apoptosis and DNA damage affected by hsa_circ_0007919 silencing (Fig. 5H-N, S3E). These results revealed that hsa_circ_0007919 increases LIG1 expression to promote cell proliferation and reduce apoptosis and DNA damage.

### hsa_circ_0007919 binds to FOXA1 and TET1 to promote LIG1 transcription

**Fig. 6:**
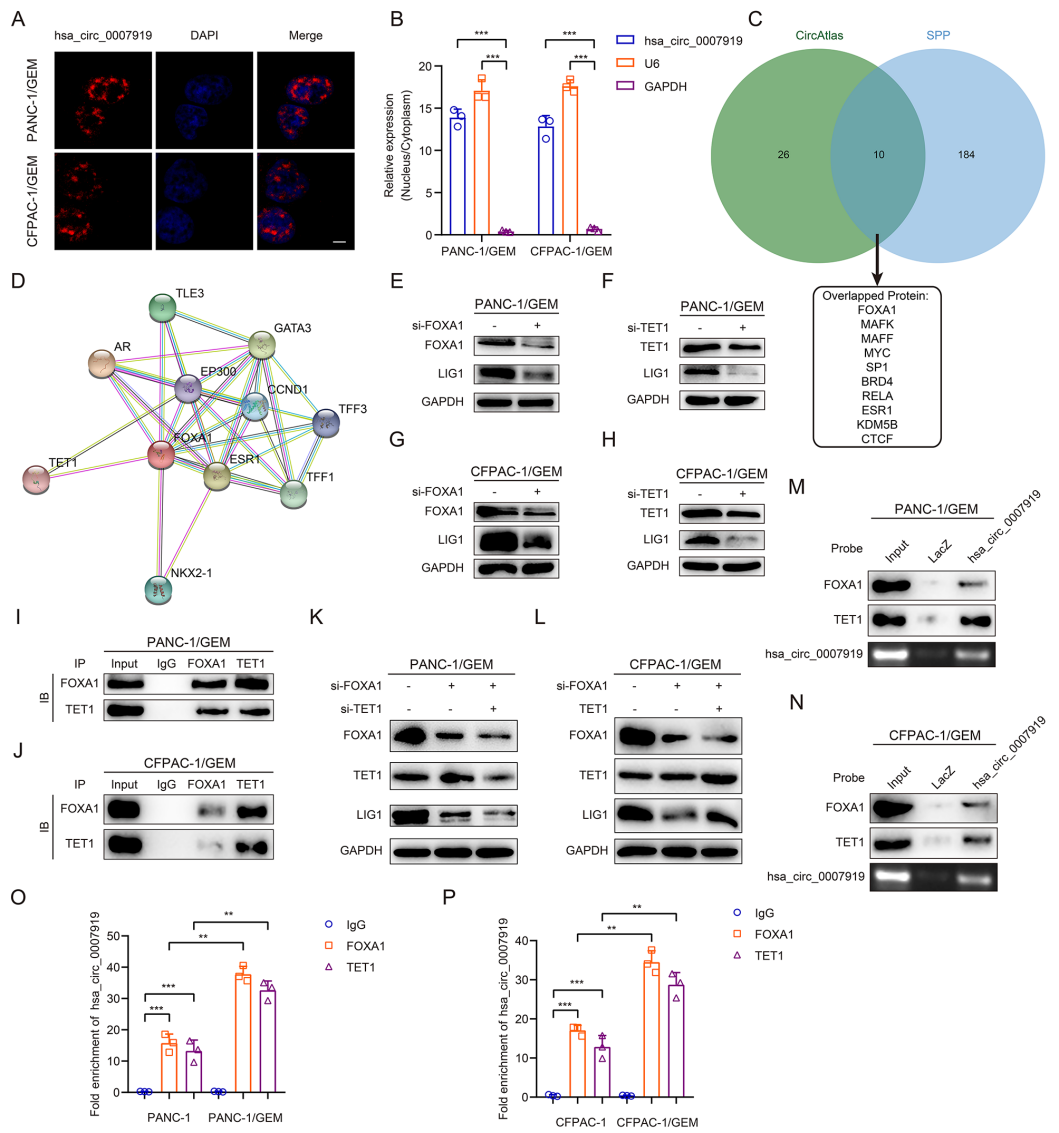
hsa_circ_0007919 binds FOXA1 and TET1 in GEM-resistant PDAC cells **(A)** FISH analysis of the cellular localization of hsa_circ_0007919. The hsa_circ_0007919 probes were red while nuclei were stained with DAPI (1000×). **(B)** Nuclear-cytoplasmic fractionation assay analysis of hsa_circ_0007919 location in GEM-resistant PDAC cells, the U6 and GAPDH were used as nuclear and cytoplasmic controls. **(C)** Venn diagram showing overlapped genes between interacting with hsa_circ_0007919 and interacting with LIG1 promoter region. **(D)** Protein-protein interaction network analysis of proteins interact with FOXA1. **(E-H)** Expression of LIG1 in GEM-resistant PDAC cells with or without FOXA1 or TET1 inhibition. **(I-J)** IP assay analysis of the interaction between FOXA1 and TET1 in GEM-resistant PDAC cells. **(K-L)** Expression of LIG1 in FOXA1-silenced GEM-resistant PDAC cells with TET1 inhibition or overexpression. **(M-N)** ChIRP assay analysis of the interaction between hsa_circ_0007919 and FOXA1 or TET1 in GEM-resistant PDAC cells. **(O-P)** RIP assay analysis of the interaction between FOXA1 or TET1 and hsa_circ_0007919 in normal or GEM-resistant PDAC cells. Data are the means ± SDs (n = 3 independent experiments), ** *p* < 0.01, *** *p* < 0.001

To investigate how hsa_circ_0007919 increases the expression of LIG1, we performed FISH and nuclear-cytoplasmic RNA fractionation assays, and the results showed that hsa_circ_0007919 was mainly distributed in the nucleus (Fig. 6A-B). We determined the overlap between the proteins that bind to hsa_circ_0007919 and the proteins that bind to LIG1 mRNA using the circAtlas 2.0 (http://circatlas.biols.ac.cn/) and ENCORI (http://starbase.sysu.edu.cn/) databases but could not identify any overlapping proteins (Fig. S4A). Then, we determined the overlap between the proteins that bind to hsa_circ_0007919 and those that bind to the promoter of LIG1 using the circAtlas 2.0 and SPP (https://www.signalingpathways.org) databases, and FOXA1 was identified as the protein with the most significant overlap (Fig. 6C). We further predicted that there may be other proteins that function together with FOXA1 and identified TET1, which binds to FOXA1, using the STRING database (https://cn.string-db.org/) (Fig. 6D). Since FOXA1 functions as a transcriptional promoter in multiple kinds of cancers and TET1 functions as a DNA methylhydroxylase to decrease the methylation level of various gene promoters and enhance their transcription [20, 21], we predicted that hsa_circ_0007919 binds to FOXA1 and TET1 to promote the transcription of LIG1. We first silenced FOXA1 and TET1 and found that the expression of LIG1 was decreased (Fig. 6E-H, S4B-C), and the results of the co-IP assay confirmed the interaction between FOXA1 and TET1 in GEM-resistant cells (Fig. 6I-J). At the same time, we silenced TET1 in FOXA1-silenced GEM-resistant cells and found that TET1 could enhanced the inhibition ability of FOXA1-silencing on LIG1, while overexpressing TET1 could partly reverse the inhibition ability of FOXA1-silencing on LIG1, which indicated that FOXA1 and TET1 play the synergistic effect in the regulation of LIG1 (Fig. 6K-L). Then, we performed a ChIRP assay and found that hsa_circ_0007919 could bind to FOXA1 and TET1 (Fig. 6M-N). Furthermore, we used a RIP assay to confirm that FOXA1 and TET1 can interact with hsa_circ_0007919, and this interaction was enhanced in GEM-resistant cells (Fig. 6O-P).

**Fig. 7:**
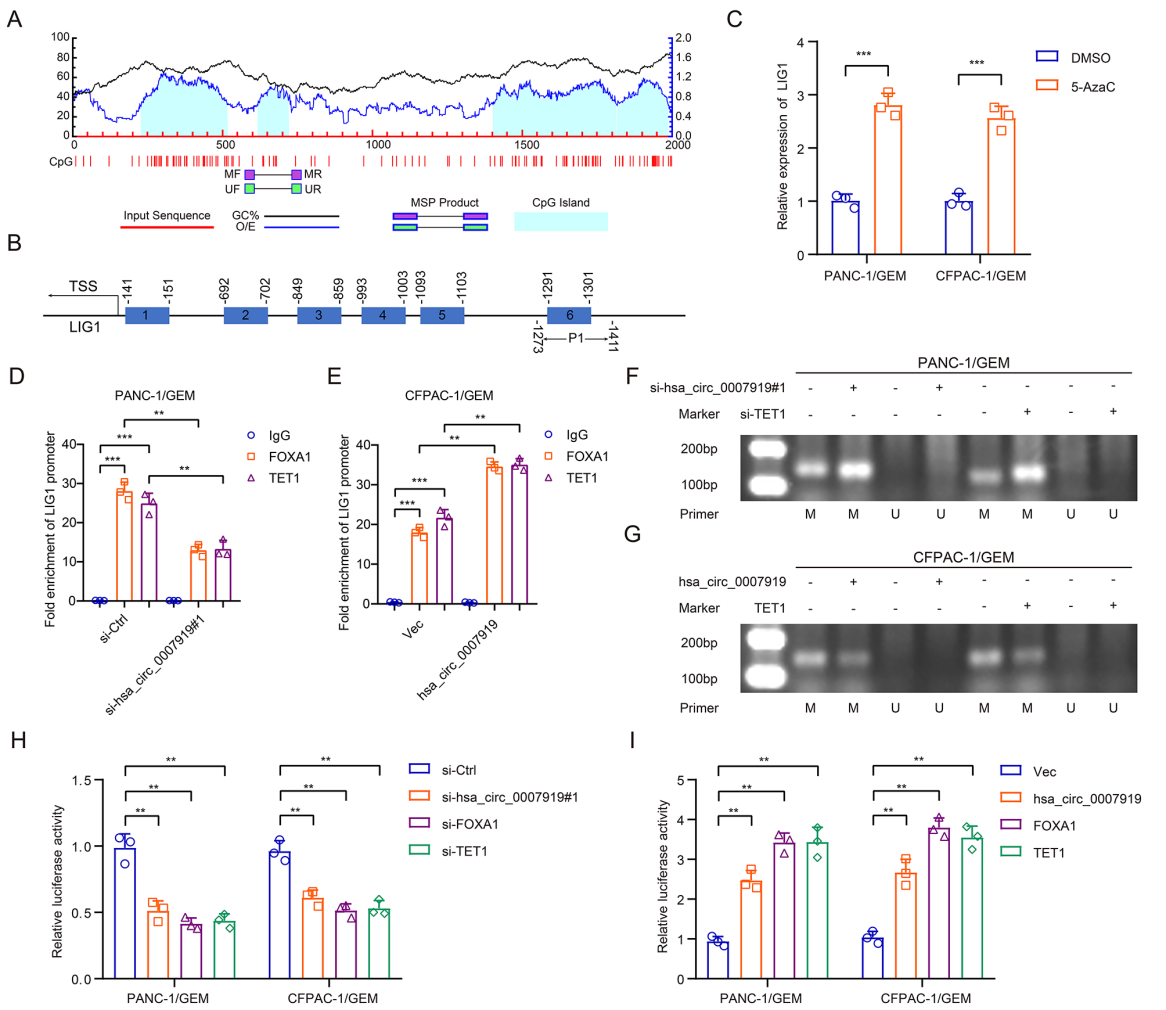
hsa_circ_0007919 recruits FOXA1 and TET1 to promote LIG1 transcription **(A)** DNA methylation analysis of CpG island of LIG1 promoter. **(B)** Interaction region predicted between FOXA1 and LIG1 promoter. **(C)** Expression of LIG1 in GEM-resistant PDAC cells with or without 5-AzaC treatment. **(D-E)** ChIP assay analysis of the interaction between FOXA1 or TET1 and LIG1 promoter. **(F-G)** MS-PCR analysis of methylation level of LIG1 promoter in GEM-resistant PDAC cells with or without hsa_circ_0007919 or TET1 inhibition and with or without hsa_circ_0007919 or TET1 overexpression. **(H-I)** Luciferase activity analysis of LIG1 transcriptional activity in GEM-resistant PDAC cells with or without hsa_circ_0007919 or FOXA1 or TET1 inhibition and with or without hsa_circ_0007919 or FOXA1 or TET1 overexpression. Data are the means ± SDs (n = 3 independent experiments), ** *p* < 0.01, *** *p* < 0.001

To investigate the interaction among FOXA1, TET1 and the LIG1 promoter, we analyzed the binding site between FOXA1 and the LIG1 promoter using the JASPAR database (https://jaspar.genereg.net/) and predicted the CpG islands in the LIG1 promoter using the MethPrimer 2.0 database (http://www.urogene.org/methprimer2/). Among the 6 sites identified by JASPAR and the 4 predicted CpG islands, we found that site 6 in the LIG1 promoter was the most enriched and thus chose the − 1411 to -1273 region (P1) for further research (Fig. 7A-B, S4D-E), and we found that treatment with 5-AzaC increased LIG1 expression in GEM-resistant cells (Fig. 7C). The results of the ChIP assay revealed that FOXA1 and TET1 bind to the LIG1 promoter region P1, and hsa_circ_0007919 inhibition decreased this binding capacity (Fig. 7D). The MS-PCR assay results showed that silencing hsa_circ_0007919 or TET1 increased the DNA methylation level in the LIG1 promoter (Fig. 7F), while overexpressing hsa_circ_0007919 or TET1 had the opposite effect (Fig. 7E and G). Furthermore, we performed a luciferase reporter assay and found that silencing hsa_circ_0007919, FOXA1 or TET1 decreased the transcriptional activity of the LIG1 promoter, while overexpressing hsa_circ_0007919, FOXA1 or TET1 enhanced LIG1 transcriptional activity (Fig. 7H-I). These results revealed that hsa_circ_0007919 enhances the transcription of LIG1 by binding to FOXA1 and TET1.

### Gemcitabine induces hsa_circ_0007919 expression through enhancing QKI-mediated back-splicing

**Fig. 8:**
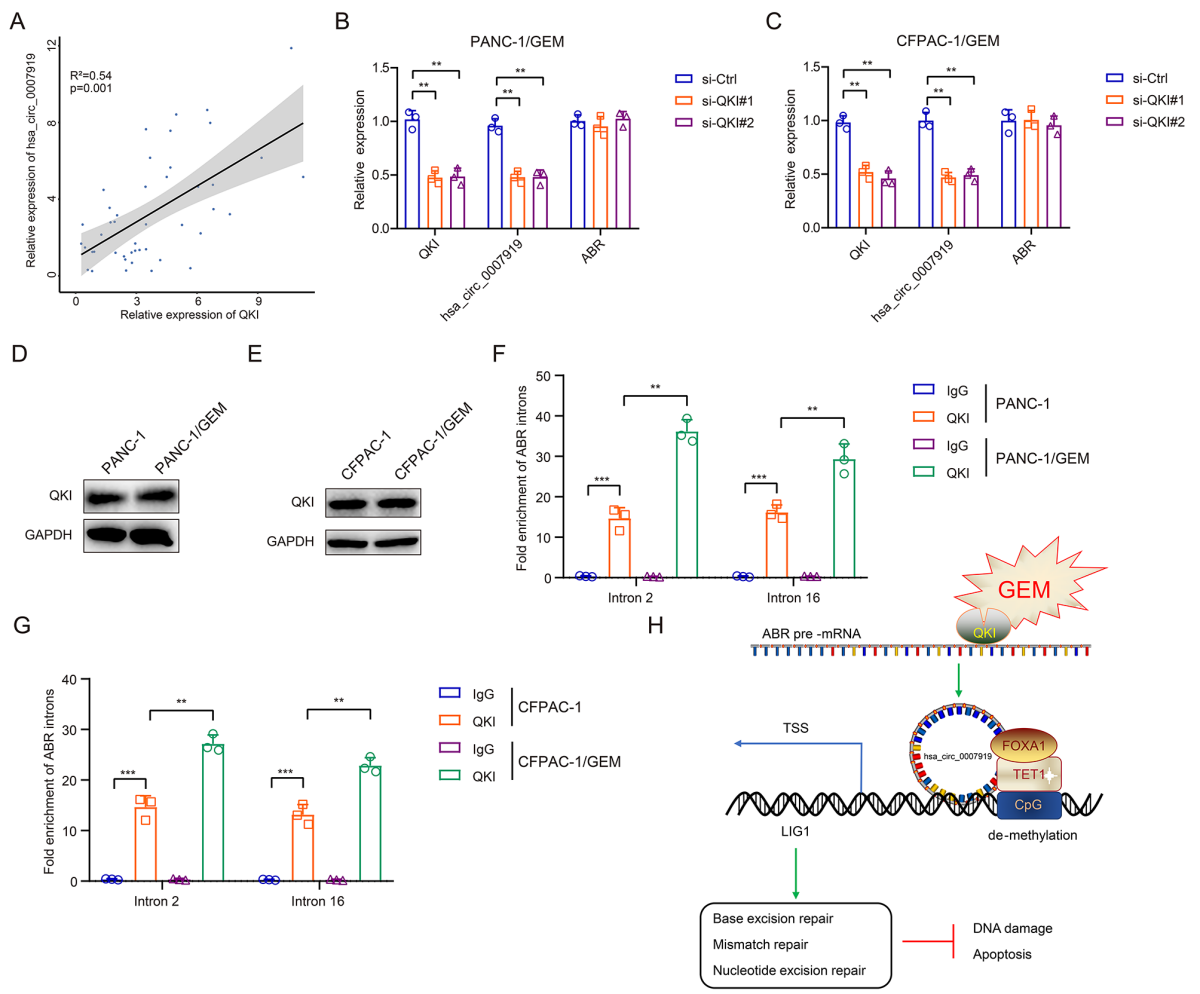
Gemcitabine induces hsa_circ_0007919 expression through enhancing QKI-mediated back-splicing **(A)** Correlation analysis of QKI and hsa_circ_0007919 expression in GEM-resistant PDAC tissues. **(B-C)** Expression of hsa_circ_0007919 and ABR in GEM-resistant PDAC cells with or without QKI inhibition. **(D-E)** Expression of QKI in normal and GEM-resistant PDAC cells. **(F-G)** RIP assay analysis of the interaction between QKI and introns of ABR pre-mRNA in normal and GEM-resistant PDAC cells. **(H)** Schematic representation showing that GEM enhances interaction between QKI and ABR pre-mRNA and promotes back-splicing and cyclization of hsa_circ_0007919. Highly expressed hsa_circ_0007919 recruits FOXA1 and TET1 to mediate de-methylation and transcription of LIG1 and upregulates the expression of LIG1. Overexpression of LIG1 activates base excision repair, mismatch repair and nucleotide excision repair pathways to inhibit the DNA damage and apoptosis of GEM-resistant PDAC cells. Data are the means ± SDs (n = 3 independent experiments), ** *p* < 0.01, *** *p* < 0.001

CircRNAs are generated by back-splicing of exons or introns of their host genes, hsa_circ_0007919 is formed by the circularization of ABR exons 3–16 (Fig. 1D), and hsa_circ_0007919 expression was found to be upregulated in GEM-resistant PDAC tissues and cells compared with normal PDAC tissues and cells (Figs. 1B and 2E). Studies have revealed that multiple proteins are involved in the process of back-splicing during circRNA synthesis. Among several well-recognized regulators, QKI and FUS were reported to enhance the formation of circRNAs, while ADAR1 was reported to exert the opposite effect [22–24]. To investigate the reason for high hsa_circ_0007919 expression, we analyzed the correlations between the expression of abovementioned proteins and that of hsa_circ_0007919 in GEM-resistant PDAC tissues and found that QKI was positively correlated with the expression of hsa_circ_0007919, while FUS had a lower correlation and ADAR1 was negatively correlated with hsa_circ_0007919 expression (Fig. 8A, S5A-B). Therefore, we predicted that QKI could promote the formation of hsa_circ_0007919. We silenced QKI in GEM-resistant PDAC cells and found that the expression of hsa_circ_0007919 was downregulated but the expression of the hsa_circ_0007919 host gene ABR was unaffected (Fig. 8B-C, S5C-D); moreover, the expression of QKI showed no difference between normal PDAC cells and GEM-resistant PDAC cells (Fig. 8D-E). QKI was reported to interact with introns flanking circRNA-formed exons in its pre-mRNA. We designed primers of ABR introns 2 and 16 and found that QKI could bind to both of these introns in PDAC cells and that this interaction was enhanced in GEM-resistant PDAC cells (Fig. 8F-G). These results revealed that GEM promotes the formation of hsa_circ_0007919 by enhancing the interaction between QKI and hsa_circ_0007919-flanking introns to promote hsa_circ_0007919 back-splicing and circularization.

In summary, this study delineates the mechanisms by which GEM enhances QKI-mediated hsa_circ_0007919 splicing and circularization and hsa_circ_0007919 recruits FOXA1 and TET1 to modulate LIG1 transcription and DNA damage repair pathways, which contribute to resistance to GEM-induced DNA damage and apoptosis in PDAC cells (Fig. 8H).

## Discussion

PDAC is one of the most aggressive and deadly malignancies and is expected to become the second leading cause of cancer-related death within a decade [2]. Although molecular mechanistic research and treatment methods for PDAC have progressed in recent decades, the 5-year survival rate of PDAC is still the lowest among all malignant tumors due to reasons including chemoresistance [25]. CircRNAs are a class of noncoding RNAs and have been identified to be involved in multiple steps in tumor development, PTK2 exon-derived hsa_circ_0005273 promotes the proliferation and metastasis of BC cells by binding to miR-200a-3p to upregulate YAP1 expression and inhibit the Hippo pathway [26], and the interaction of circ-GALNT16 with p53 is enhanced to inhibit the proliferation and metastasis of colorectal cancer cells via the inhibition of Senp2-mediated hnRNPK desumoylation [27]. We mainly focused on the relationships between circRNAs and GEM resistance in PDAC. Three GEM-resistant PDAC and three GEM-sensitive PDAC tissues were collected from clinical surgical specimens for analysis of circRNA expression levels with a circRNA chip, and hsa_circ_0007919 expression was found to be significantly increased in GEM-resistant PDAC tissues. Hsa_circ_0007919 is located on chr17:953289–1,003,975, with a length of 1545 bp. We found that hsa_circ_0007919 was highly expressed in GEM-resistant PDAC tissues and cells, and hsa_circ_0007919 inhibited apoptosis and DNA damage induced by GEM treatment. It has been shown that hsa_circ_0007919 is involved in the progression of ulcerative colitis and tuberculosis [28, 29], and the current study suggests that hsa_circ_0007919 plays an important role in GEM resistance in PDAC.

The predominant cancer treatments, other than surgery, are radiation and chemotherapy, which act by inducing DNA damage [30]. GEM is a common drug used in clinical chemotherapy for PDAC and usually acts by inducing SSBs [8], and a DSB can be formed when two SSBs are located near each other or when the DNA replication apparatus encounters SSBs, while DSBs are difficult to repair and extremely toxic [31]. To combat the hazard posed by DNA damage, cancer cells have evolved mechanisms called DDR pathways to facilitate DNA damage repair [32]. Among the components of these pathways, LIG1, a DNA ligase, completes the repair of almost all types of DNA damage by religating the broken phosphodiester skeleton in DSBs [33]. In addition, genetic deletion or low expression of LIG1 was found to be associated with selective carboplatin resistance in preclinical models of triple-negative breast cancer (TNBC)[34]. LIG1 deletion in ovarian cancer (OC) cells increased platinum cytotoxicity, which was associated with the accumulation of DSBs, S-phase arrest and increased proportions of apoptotic cells [19]. We performed RNA-seq analysis in control and hsa_circ_0007919-silenced GEM-resistant PDAC cells, and KEGG enrichment analysis and GSEA were performed on the identified differentially expressed genes. The results indicated that base excision repair, mismatch repair and nucleotide excision repair were the top-ranked enriched pathways, and LIG1 was enriched in all three DNA damage repair pathways. Here, we found that silencing hsa_circ_0007919 decreased LIG1 expression and inhibited LIG1-mediated multiple DNA damage repair pathways to develop resistant to GEM in GEM-resistant PDAC cells. These results indicate that hsa_circ_0007919 could be a potential therapeutic target for GEM-resistant PDAC treatment.

FOXA1 is a member of the Forkhead Box protein family that is involved in cell growth and differentiation and is also a DNA binding protein involved in transcription and DNA repair [35]. Many members of the Forkhead Box protein family are associated with pancreatic metabolism and differentiation and the development of pancreatic cancer (PC), FOXO1 inhibition can mimic β-cell differentiation by downregulating β-cell-specific transcription and lead to abnormal expression of progenitor genes and the α-cell marker glucagon [36]. FOXD1 directly promotes the transcription of SLC2A1 and inhibits the degradation of SLC2A1 through the RNA-induced silencing complex, thus promoting aerobic glycolysis in PC cells and enhancing their proliferation and metastasis [37]. Meanwhile, FOXA1 was reported to be associated with multiple kinds of cancers, especially prostate cancer (PCa) and BC. FOXA1 contributes to the activation of androgen receptor (AR) signaling that drives the growth and survival of PCa cells through direct interaction with AR and also has an AR-independent role in regulating epithelial-mesenchymal transition (EMT)[38], FOXA1 binds to the DNA-binding domain of STAT2 and inhibits STAT2 DNA-binding activity, IFN signaling gene expression and the tumor immune response in PCa and BC [39]. In addition to the binding of transcription factors to DNA promoter regions, methylation of DNA promoter regions also plays an important role in gene expression regulation, with hypermethylation of most gene promoter regions leading to reduced transcription levels [40]. TET1, a DNA demethylase, maintains genomic methylation homeostasis and accomplishes epigenetic regulation, which affect stem cells, immune responses and various malignant tumors [41]. TET1 promotes the transcription of CHL1 by binding to and demethylating the CHL1 promoter, thereby inhibiting the Hedgehog pathway, inhibiting EMT and sensitizing PDAC cells to 5-FU and GEM [42]. However, the role of FOXA1 and TET1 on GEM resistance in PDAC remains unknown. Here, we found that FOXA1 and TET1 can both bind to the promoter of LIG1 and that TET1 mediates demethylation of the LIG1 promoter and enhances FOXA1-mediated transcription of LIG1. It has been reported that FOXA2, a transcription factor precursor, was required for the regulation of pancreatic endoderm development, and TET1 deletion results in significant changes in FOXA2 binding in pancreatic progenitor cells. Loci with reduced FOXA2 binding have a low level of active chromatin modification and enrichment of bHLH motifs, resulting in functional β-cell defects [43]. In this study, we also confirmed that TET1 could increase the transcriptional activity of FOXA1 in GEM-resistant PDAC cells, which similar to the interaction between TET1 and FOXA2. These results enriched the further understanding of the interaction between DNA demethylase and transcription factors and the synergistic effect of transcriptional regulation.

CircRNAs are generated by back-splicing of pre-mRNAs produced by transcription of host genes, and cis-regulatory elements, trans-acting factors, RNA binding proteins (RBPs) and other related molecules can regulate the splicing and circularization of circRNAs [44]. Among these regulatory factors, QKI belongs to the STAR family containing KH domain RNA-binding proteins and has been found to affect pre-mRNA splicing, and QKI binds up- and down-stream of the circRNA-forming exons in SMARCA5 to promote circRNA formation [45]. FUS is a member of the FET protein family and is reported to be a regulator of circRNA biogenesis; CircROBO1 upregulates KLF5 by sponging miR-217-5p, enabling KLF5 to activate FUS transcription and promote circROBO1 back-splicing, forming a positive feedback loop to enhance BC-derived liver metastasis [46]. ADAR1 is a member of the ADAR enzyme family that facilitates A-to-I editing of RNAs, circNEIL3 can inhibit ADAR1 expression by inducing GLI1 RNA editing through sponging miR-432-5p, and ADAR1 inhibition increases circNEIL3 expression to promote EMT and cell cycle progression in PDAC [47]. We found that GEM treatment enhances the interaction between QKI and introns 2 and 16 of ABR pre-mRNA to promote the splicing and circularization of hsa_circ_0007919. These results suggest that GEM treatment situation could enhance the function of QKI without changing its expression, which indicates a critical adaptation mechanism to developing resistance to GEM or other chemotherapy agents.

Taken together, our findings indicate that hsa_circ_0007919 can promote DNA damage repair to confront GEM treatment. Mechanistically, hsa_circ_0007919 recruits FOXA1 and TET1 to promote LIG1 transcription and activates the base excision repair, mismatch repair and nucleotide excision repair pathways to ameliorate the DNA damage and suppress the apoptosis induced by GEM. Furthermore, GEM treatment-enhanced interaction between QKI and ABR pre-mRNA led to increased biogenesis of hsa_circ_0007919 in a back-splicing-dependent manner. Our findings could be helpful for understanding the mechanism of GEM resistance and developing therapeutic strategies for chemotherapy-resistant PDAC.

### Electronic supplementary material

Below is the link to the electronic supplementary material.

Supplementary Material 1

Supplementary Material 2

Supplementary Material 3

Supplementary Material 4

Supplementary Material 5

## Data Availability

The data generated in this study are available upon request from the corresponding author.

## Abbreviations

circRNA: Circular RNA
GEM: Gemcitabine
PDAC: Pancreatic ductal adenocarcinoma
OS: Overall survival
DFS: Disease-free survival
DDR: DNA damage response
5-FU: 5-fluorouracil
BC: Breast cancer
FBS: Fetal Bovine Serum
IF: Immunofluorescence
BSA: Bovine Serum Albumin
IHC: Immunohistochemical
GSEA: Gene set enrichment analysis
FISH: Fluorescence in situ hybridization
ChIRP: Chromatin Isolation by RNA Purification
Co-IP: Co-immunoprecipitation
RIP: RNA binding protein immunoprecipitation
ChIP: Chromatin immunoprecipitation
MS-PCR: Methylation-specific PCR
SD: Standard deviation
SSB: Single-strand break
DSB: Double-strand break
TNBC: Triple-negative breast cancer
OC: Ovarian cancer
PC: Pancreatic cancer
PCa: Prostate cancer
AR: Androgen receptors
EMT: Epithelial-interstitial transition
RBP: RNA binding protein
