# Correction: hsa_circ_0007919 induces LIG1 transcription by binding to FOXA1/TET1 to enhance the DNA damage response and promote gemcitabine resistance in pancreatic ductal adenocarcinoma

**DOI:** 10.1186/s12943-024-01937-9

**Published:** 2024-01-16

**Authors:** Lei Xu, Xiao Ma, Xiuzhong Zhang, Chong Zhang, Yi Zhang, Shuai Gong, Nai Wu, Peng Zhang, Xinyu Feng, Jiaxuan Guo, Mengmeng Zhao, Zeqiang Ren, Pengbo Zhang

**Affiliations:** 1 Department of General Surgery, Affiliated Hospital of Xuzhou Medical University, Xuzhou, China; 2 Institute of Digestive Diseases, Xuzhou Medical University, Xuzhou, China; 3 Shandong First Medical University and Shandong Academy of Medical Sciences, Shandong Cancer Hospital and Institute, Jinan, China; 4 Department of General Surgery, Xuzhou First People’s Hospital, Xuzhou, China; 5 Department of General Surgery, Shangqiu Municipal Hospital, Shangqiu, China

**Correction: Mol Cancer 22, 195 (2023)**

**https://doi.org/10.1186/s12943-023-01887-8**

Following publication of the original article [1], the authors found that the grouping tags in Fig. 1A was mislabeled and led to completely opposite representations, so they would like to correct the picture. The correct figure is given below.

**Fig. 1:**
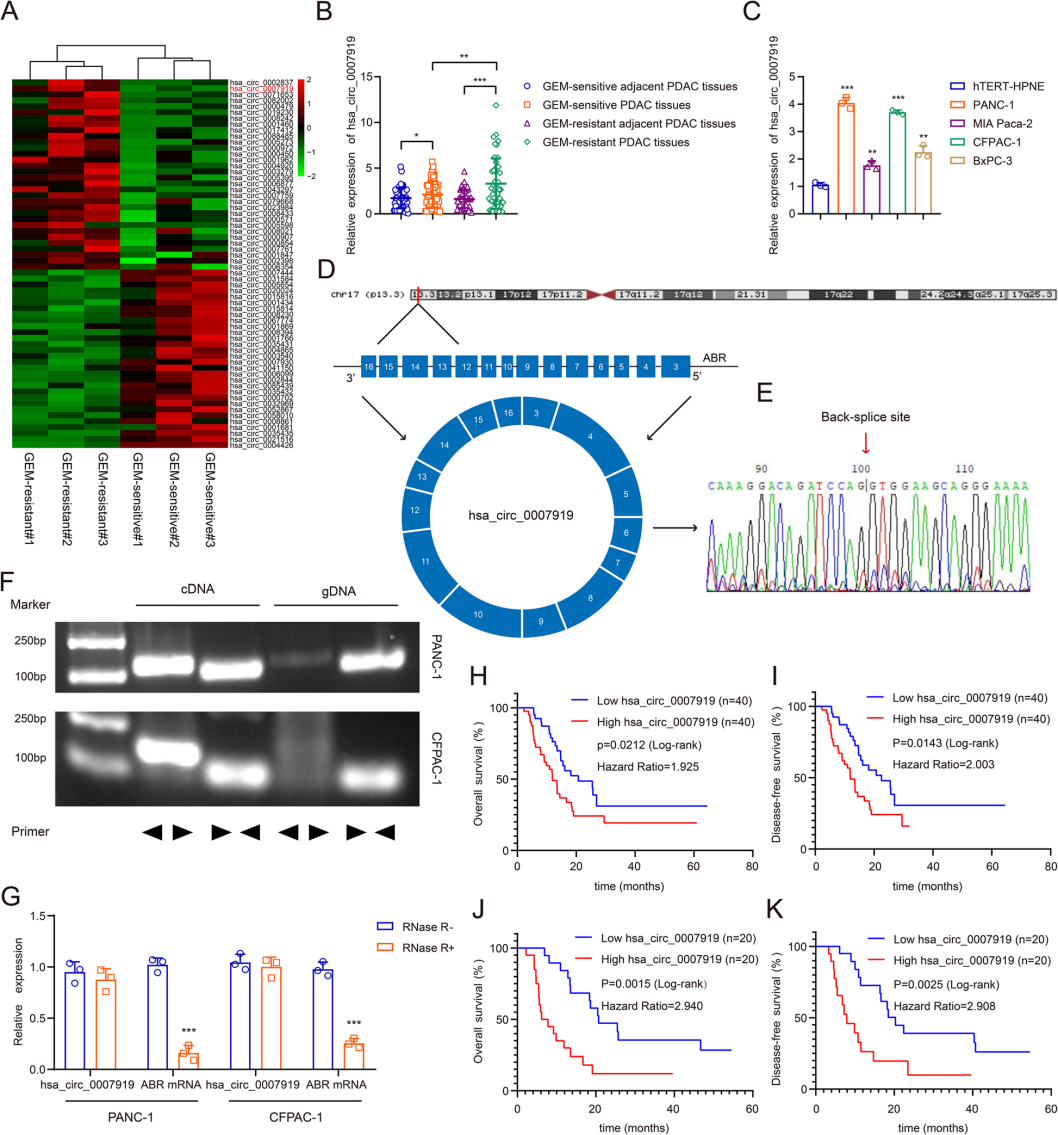
hsa_circ_0007919 is upregulated in GEM-resistant PDAC and predicts poor prognosis. **(A)** Hierarchical clustering showing differentially expressed circRNAs in GEM-sensitive and GEM-resistant PDAC tissues (FC > 1 or < -1, *p* < 0.05). **(B)** The relative expression of hsa_circ_0007919 in GEM-sensitive and GEM-resistant PDAC tissues and corresponding adjacent PDAC tissues. **(C)** The relative expression of hsa_circ_0007919 in PDAC cells and normal pancreatic cells. **(D)** The genomic location and back-splicing of hsa_circ_0007919. **(E)** The splicing site of hsa_circ_0007919 validated by Sanger-seq. **(F)** PCR and agarose gel electrophoresis analysis of the presence of hsa_circ_0007919 and ABR in cDNA and gDNA samples from PDAC cells. **(G)** Expression of hsa_circ_0007919 and ABR in PDAC cells with or without RNase R treatment. **(H-I)** Kaplan–Meier analysis of the OS rate and DFS rate in PDAC patients with high or low expression of hsa_circ_0007919. **(J-K)** Kaplan–Meier analysis of the OS rate and DFS rate in GEM-resistant PDAC patients with high or low expression of hsa_circ_0007919. Data are the means ± SDs (n = 3 independent experiments), * *p* < 0.05, ** *p* < 0.01, *** *p* < 0.001
