# Supplementary figures and images for: hsa_circ_0007919 induces LIG1 transcription by binding to FOXA1/TET1 to enhance the DNA damage response and promote gemcitabine resistance in pancreatic ductal adenocarcinoma
Source: Mol Cancer. 2023 Dec 4;22:195. doi: 10.1186/s12943-023-01887-8 (PMC10694898; doi:10.1186/s12943-023-01887-8)

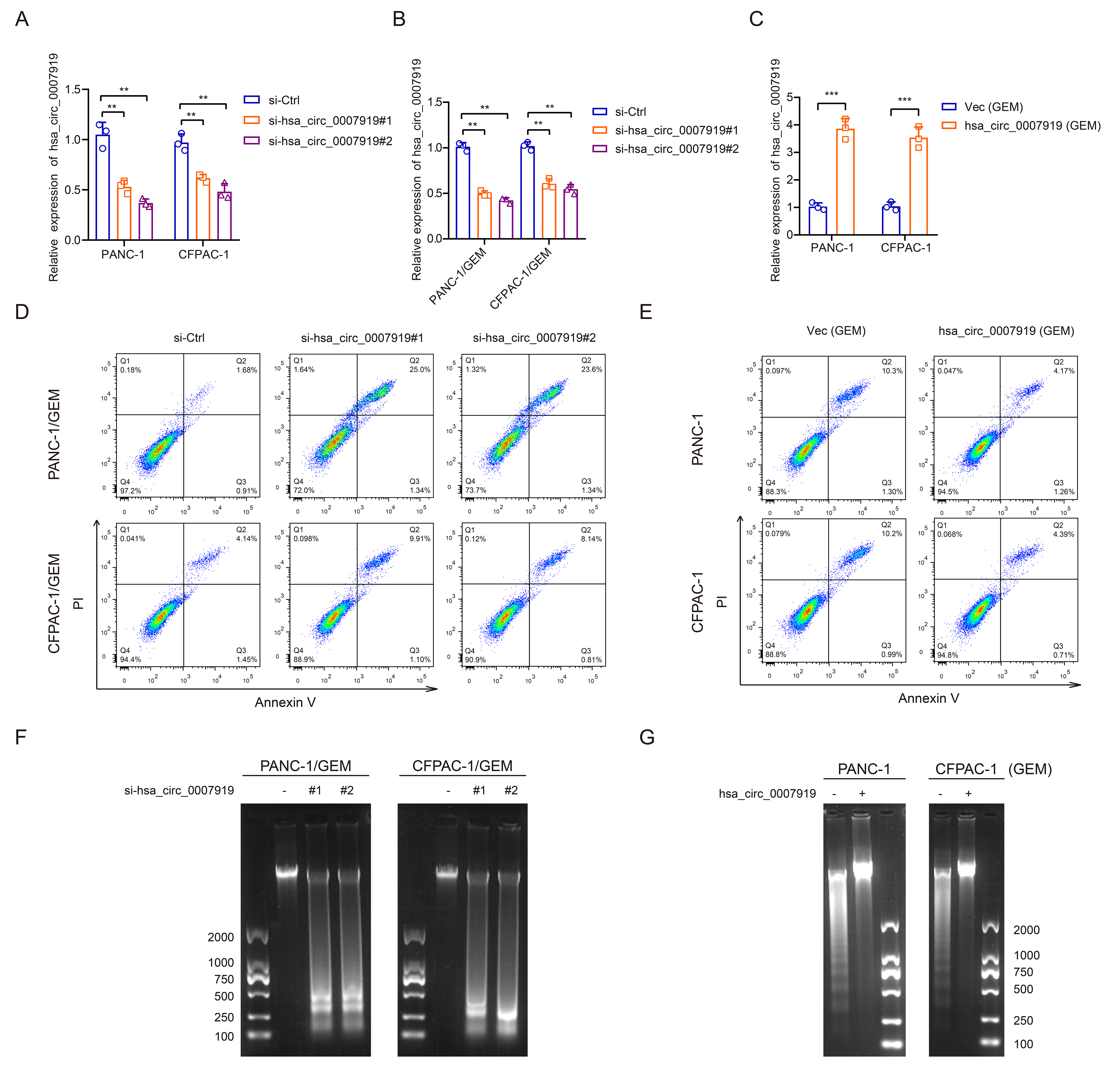

Supplement: Supplementary file 1 — Supplementary Material 1 [file 12943_2023_1887_MOESM1_ESM.png]

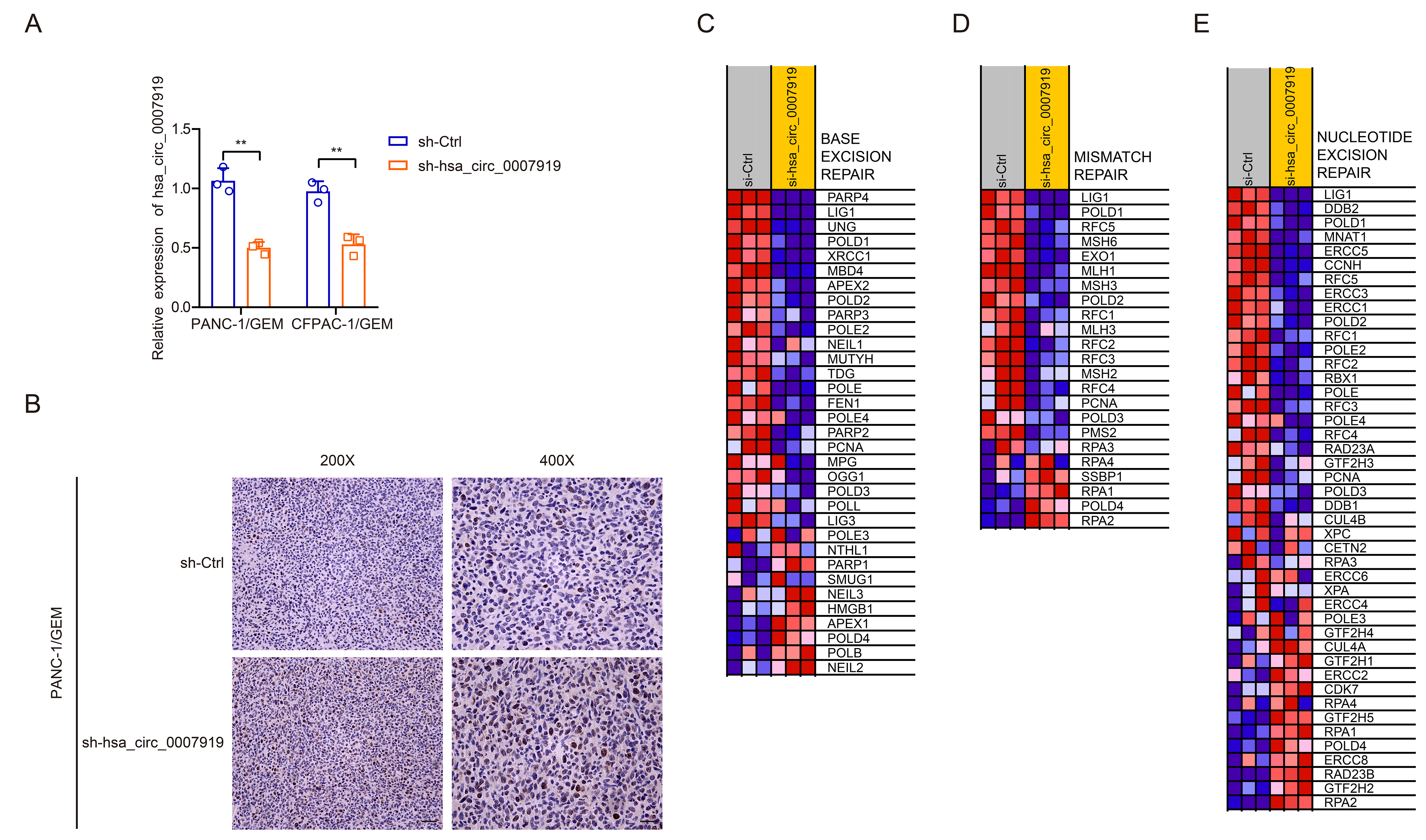

Supplement: Supplementary file 2 — Supplementary Material 2 [file 12943_2023_1887_MOESM2_ESM.png]

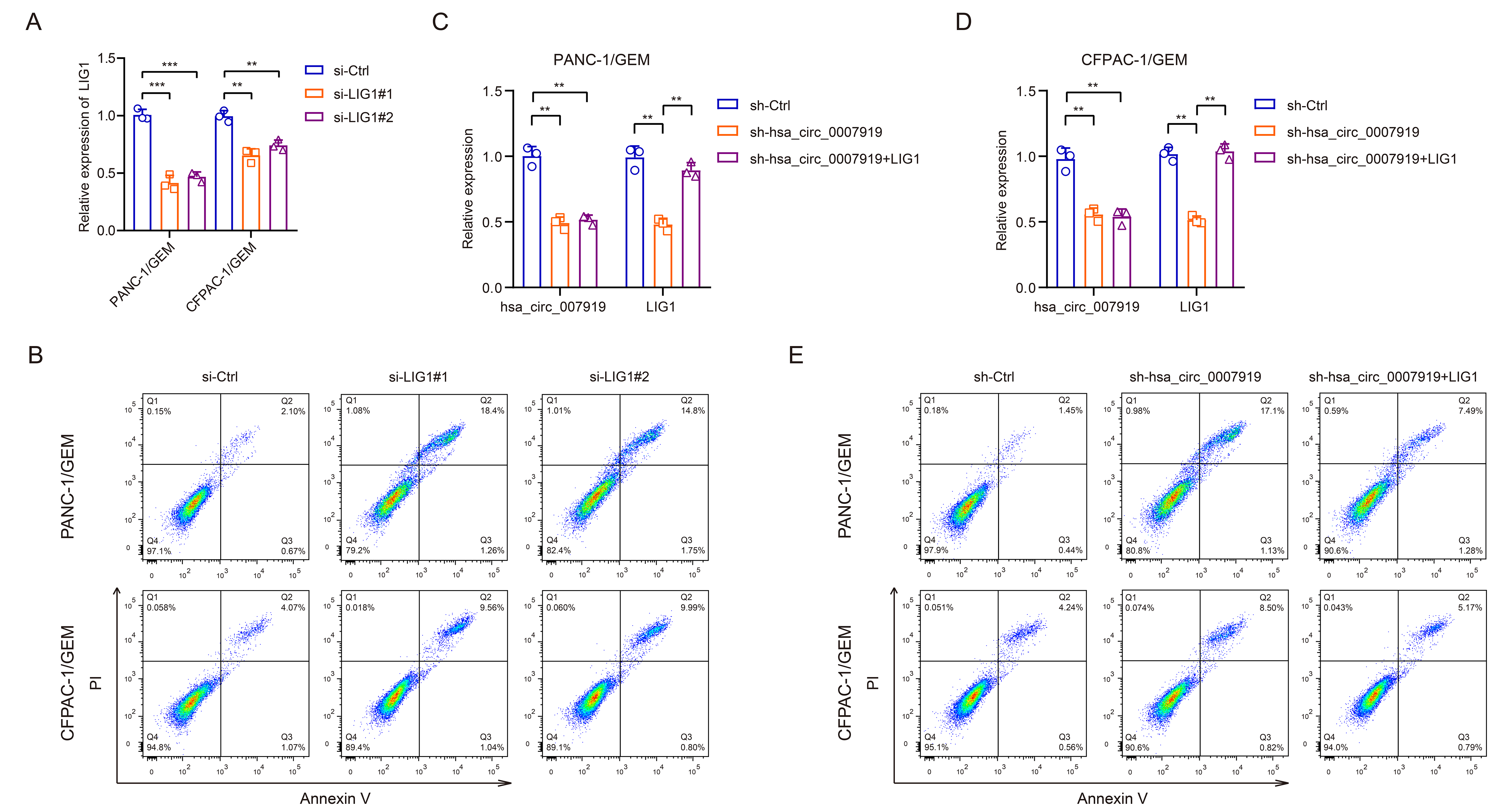

Supplement: Supplementary file 3 — Supplementary Material 3 [file 12943_2023_1887_MOESM3_ESM.png]

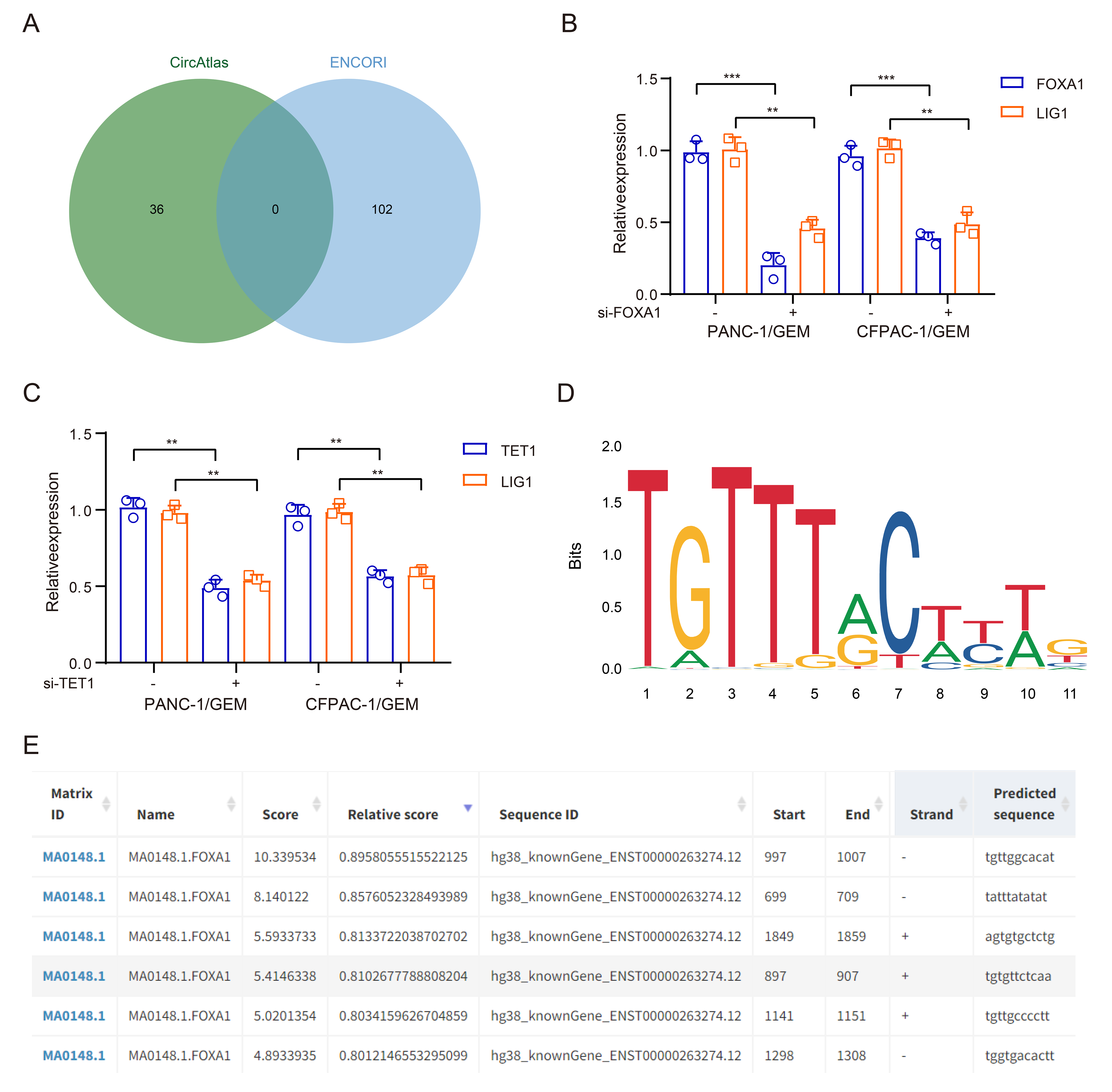

Supplement: Supplementary file 4 — Supplementary Material 4 [file 12943_2023_1887_MOESM4_ESM.png]

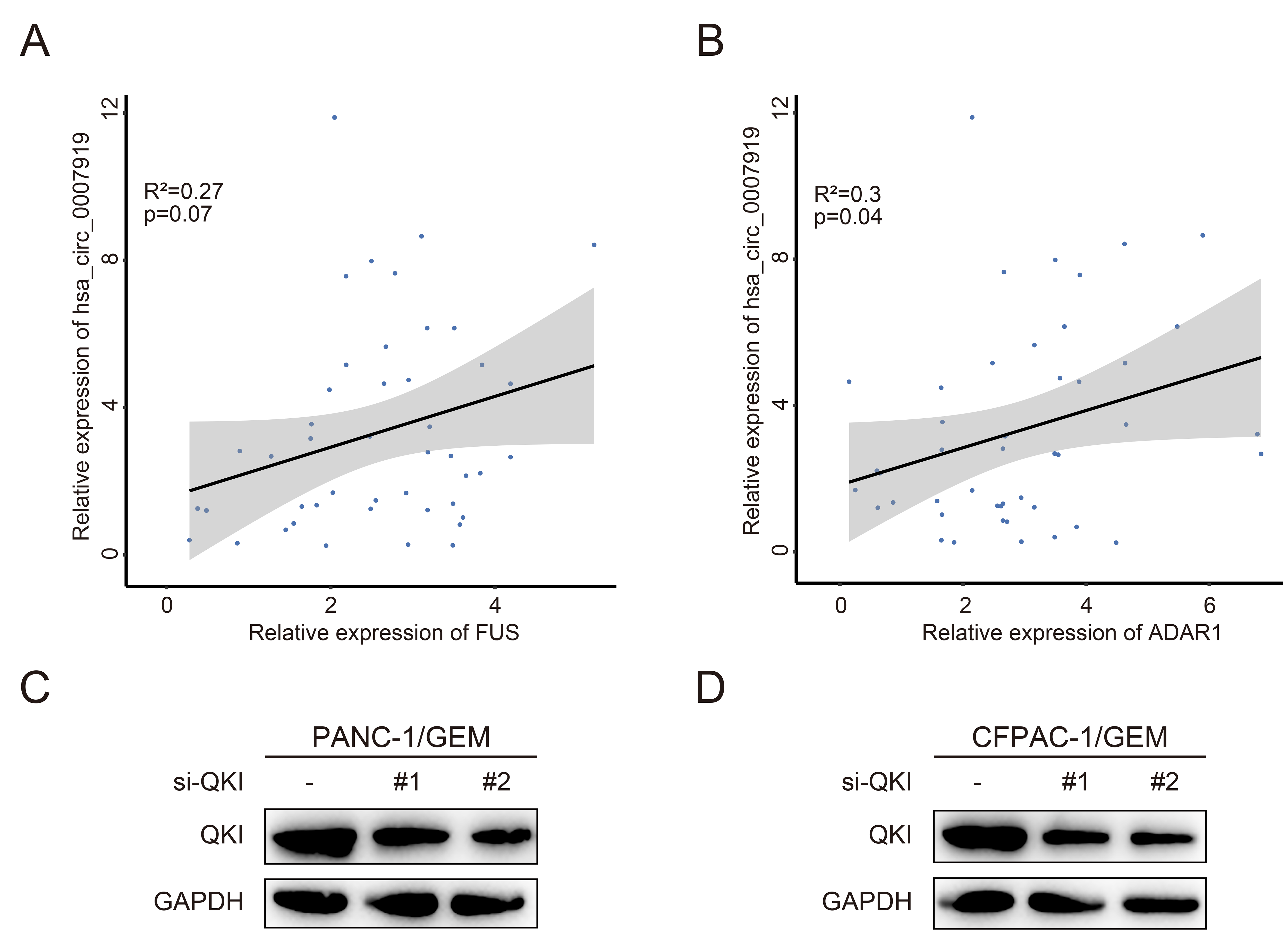

Supplement: Supplementary file 5 — Supplementary Material 5 [file 12943_2023_1887_MOESM5_ESM.png]
